# Understanding the variability of Australian fire weather between 1973 and 2017

**DOI:** 10.1371/journal.pone.0222328

**Published:** 2019-09-19

**Authors:** Sarah Harris, Chris Lucas

**Affiliations:** 1 Bushfire Management, Country Fire Authority, Burwood East, Victoria, Australia; 2 School of Earth, Atmosphere and Environment, Monash University, Clayton, Victoria, Australia; 3 Science to Services, Bureau of Meteorology, Melbourne, Victoria, Australia; Woods Hole Oceanographic Institution, UNITED STATES

## Abstract

Australian fire weather shows spatiotemporal variability on interannual and multi-decadal time scales. We investigate the climate factors that drive this variability using 39 station-based historical time series of the seasonal 90^th^-percentile of the McArthur Forest Fire Danger Index (FFDI) extending from 1973 through 2017. Using correlation analyses, we examine the relationship of these time series to the El Niño Southern Oscillation (ENSO), the Southern Annular Mode (SAM) and the Indian Ocean Dipole (IOD), considering both concurrent and time-lagged relationships. Additionally, longer term behaviour of the time series using linear trend analysis is discussed in the context of the climate drivers, Interdecadal Pacific Oscillation (IPO) and anthropogenic climate change. The results show that ENSO is the main driver for interannual variability of fire weather, as defined by FFDI in this study, for most of Australia. In general, El Niño-like conditions lead to more extreme fire weather, with this effect stronger in eastern Australia. However, there are significant regional variations to this general rule. In NSW, particularly along the central coast, negative SAM is a primary influence for elevated fire weather in late-winter and spring. In the southeast (VIC and TAS), the El Niño-like impact is exacerbated when positive IOD conditions are simultaneously observed. The spring conditions are key, and strongly influence what is observed during the following summer. On longer time scales (45 years), linear trends are upward at most stations; this trend is strongest in the southeast and during the spring. The positive trends are not driven by the trends in the climate drivers and they are not consistent with hypothesized impacts of the IPO, either before or after its late-1990s shift to the cold phase. We propose that anthropogenic climate change is the primary driver of the trend, through both higher mean temperatures and potentially through associated shifts in large-scale rainfall patterns. Variations from interannual factors are generally larger in magnitude than the trend effects observed to date.

## Introduction

Wildfires, or bushfires as they are more commonly known in Australia, can have devastating consequences when intersecting with society values. When and where a bushfire occurs is related to four 'switches': 1) ignition, either human-caused or from natural sources; 2.) fuel abundance and continuity–a sufficient amount of fuel must be present; 3.) fuel dryness, with lower moisture content leading to a higher chance for fire; and 4.) suitable 'fire weather' conditions for fire spread, generally hot, dry and windy [1]. The state of these switches is strongly dependent on the meteorological conditions across multiple spatiotemporal scales, ranging from short and local (e.g. a stand of trees over a few minutes) to planet-sized variations in oceanic and atmospheric circulations [1]. Fire weather is also expected to change in Australia (and elsewhere) as anthropogenic climate change manifests itself [2].

Fire weather can, for some areas, be summarised by a fire weather index, a combination of different meteorological factors and fuel information of relevance to the risks associated to wildfires [3]. Fire weather indices are typically expressed through some combination of surface air temperature, precipitation, relative humidity and wind speed [4]. A commonly used index in Australia is the McArthur’s Forest Fire Danger Index (FFDI). This index combines weather variables to determine expected fire behaviour for open canopy dry Eucalypt forest in eastern Australia [5]. The index was originally developed to determine the difficulty of suppressing a fire but is now used more broadly to issue warnings by the fire agencies. More severe fire weather, as captured by FFDI, during a bushfire event typically results in greater than average numbers of houses and/or lives lost in historical Australian fires [6,7].

In this study, we are concerned with changes across Australia over seasonal, interannual and longer-term time scales. At these scales, Australian climate variability is driven by several modes of variability that result in spatiotemporal variations of temperature and rainfall [8,9]. Logically, these modes should also impact the variability of Australian fire weather, but the relationships are not entirely clear. Some of the most significant climate drivers for Australia are the El Niño Southern Oscillation (ENSO) [10,11], Indian Ocean Dipole (IOD) [12], and the Southern Annular Mode (SAM) [13]. These climate drivers involve changes in the ocean and atmosphere that can be both near Australia and remote from Australia and affect different parts of the continent in different ways and at various times of the year.

Multiple studies have found linkages between these climate drivers and FFDI, for various regions of Australia. Most widely studied has been ENSO, with El Niño years resulting in higher fire dangers [3,14–17] and increased fire activity [18–22]. For the IOD, the positive phase occurring in winter and spring has been linked to higher fire danger over southeastern Australia [23,24]. Finally, SAM has been identified as a controlling factor of interannual and century-scale fire activity in Tasmania [25]. However, there are no studies that analyse the independent and co-dependent influences of these climate drivers on fire weather for the whole of Australia.

In addition to this interannual variability, the climate of Australia is changing over longer time scales. The *State of the Climate 2018* report for Australia [26] notes that the average surface temperatures have increased by just over 1.0° C since 1910, with about 70% of this change occurring since the 1970s. There has also been about a 10–20% decline in cool season rainfall across southern Australia since the 1970s, while many parts of northern Australia have seen wetter conditions. Coincident with these changes, an increasing trend in seasonal values of FFDI has been noted across much of southern Australia [3,4]. These changes are believed to be closely associated with the observed global increase in greenhouse gases, but the signal is potentially modulated by the Interdecadal Pacific Oscillation (IPO), an ENSO-like pattern of decadal (i.e. 10–40 year) SST variability that has been identified through statistical analysis of historical records [27] and linked to fire risk [15]. While the science of climate change continues to strengthen, Australian fire weather studies have less often investigated the link between observed increases in fire weather and climate change. However, a recent study based on gridded data across Australia found that changes in fire weather conditions in southern Australia are attributable, at least in part, to anthropogenic climate change, including in relation to increasing temperatures [3].

In this study, we will investigate the interannual and longer-term variability of fire weather, as measured by FFDI, at individual station locations across Australia for the period from 1973 through 2017 and explore the relationships and drivers of the trends and variability. Understanding the interactions between climate drivers and Australian fire weather has the potential to provide information that may result in more effective fire planning and resource management on seasonal and long term scales.

## Data and methodology

### Fire weather data

**Fig 1:**
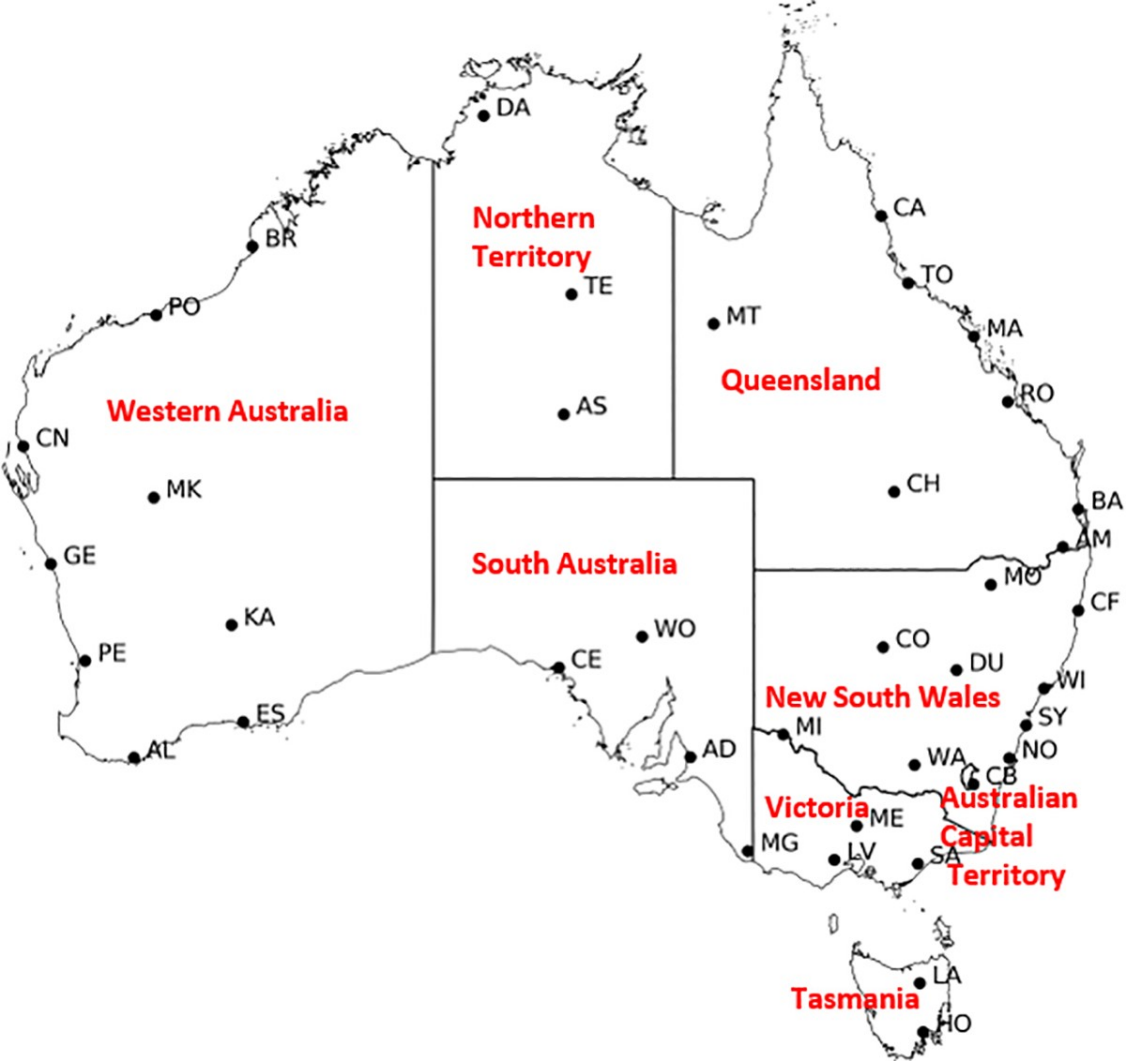
Map of 39 stations locations (for full names of stations refer to Table 1) and the states and territories labelled in red.

Fire weather data in this study come from the historical fire weather dataset described by [28]. This dataset is based on daily station-based measurements of meteorological variables across Australia (Fig 1 and Table 1). Following [4], this dataset has been extended to 2017, and thus has an additional seven years. A subset of the 39 stations with the most complete records are chosen for further analysis in this study. Fire weather in this dataset is represented by the McArthur Forest Fire Danger Index (FFDI) [5] and the numerical algorithm described in [29]. Specifically, FFDI is calculated as

FFDI=1.2753exp(0.987ln(DF)+0.0338T−0.0345RH+0.0234V)

where *DF* is the drought factor, *T* is the daily maximum temperature (°C), *RH* the 1500 local time (LT) relative humidity (%) and *V* is the 1500 LT wind speed (km h^-1^). The drought factor (*DF*) is calculated following [30] using the Keetch Byram Drought Index (KBDI) [31] as the basis for the soil moisture deficit. KBDI is computed using daily observations of rainfall taken at 0900 LT. No consideration of varying fuel types or amount or the slope of the terrain is made by FFDI. While this is a limitation when considering fire behaviour, FFDI is used here as a general proxy for the characteristics of the fire-weather climate.

**Table 1:** The list of station included in the study along with the abbreviation, state, latitude and longitude.

Station (Abbreviation)	State	Latitude	Longitude
Adelaide (AD)	SA	-34.92	138.62
Albany Airport (AL)	WA	-34.94	117.80
Alice Springs (AS)	NT	-23.80	133.89
Amberley (AM)	QLD	-27.63	152.71
Brisbane Airport (BA)	QLD	-27.39	153.13
Broome (BR)	WA	-17.95	122.23
Cairns (CA)	QLD	-16.87	145.75
Canberra (CB)	ACT	-35.30	149.20
Carnarvon (CN)	WA	-24.89	113.67
Ceduna (CE)	SA	-32.13	133.70
Charleville (CH)	QLD	-26.42	146.25
Cobar (CO)	NSW	-31.49	145.83
Coffs Harbour (CF)	NSW	-30.31	153.12
Darwin (DA)	NT	-12.42	130.89
Dubbo (DU)	SA	-32.22	148.57
Esperance (ES)	WA	-33.83	121.89
Geraldton (GE)	WA	-28.80	114.70
Hobart (HO)	TAS	-42.89	147.33
Kalgoorlie (KA)	WA	-30.78	121.45
Launceston Airport (LA)	TAS	-41.54	147.20
Laverton (LV)	VIC	-37.86	144.76
Mackay (MA)	QLD	-21.12	149.22
Meekatharra (MK)	WA	-26.61	118.54
Melbourne Airport (ME)	VIC	-37.68	144.84
Mildura (MI)	VIC	-34.23	142.08
Moree (MO)	NSW	-29.49	149.85
Mt Gambier (MG)	SA	-37.75	140.77
Mt Isa (MT)	QLD	-20.68	139.49
Nowra (NO)	NSW	-34.95	150.54
Perth Airport (PE)	WA	-31.93	115.98
Port Hedland (PO)	WA	-20.37	118.63
Rockhampton (RO)	QLD	-23.38	150.48
Sale (SA)	VIC	-38.12	147.13
Sydney Airport (SY)	NSW	-33.94	151.17
Tennant Creek (TE)	NT	-19.64	134.18
Townsville (TO)	QLD	-19.25	146.77
Wagga (WA)	NSW	-35.16	147.46
Williamtown (WI)	NSW	-32.79	151.84
Woomera (WO)	SA	-31.16	136.81

The period of analysis extends from December 1972 through June 2017. The data are further refined by considering the seasonal distributions of FFDI over the 'standard' meteorological seasons of DJF (December January February), MAM (March April May), JJA (June July August) and SON (September October November). Daily FFDI for each season and each fire year (July-June) are ranked, and the 90^th^ percentile (termed FFDI90) is identified. This variable is representative of the upper end of the fire weather spectrum for a given season. Specific FFDI90 data points are identified by the season name and the last two digits of the year; for DJF, the 'year' refers to that of January and February (i.e. DJF73 means December 1972 through February 1973).

The FFDI90 variables are homogenized to account for differences in the wind speed measurement techniques at a given station over time, following the method described by [28]. No adjustments are made for any possible temperature, humidity or rainfall inhomogeneities. Over the time of this analysis, these are expected to have a relatively small impact on the long-term stability of the data [28].

In general, there are *N* = 44 years of available data. This applies to FFDI90 during JJA and SON. One additional year (i.e. *N* = 45) is available during DJF and MAM for FFDI90. A few stations have missing seasons: Brisbane AP during DJF73, Launceston for JJA08 and Mt Isa for DJF73.

### Interpreting FFDI: Fuel, climate and fire regime

At any time of the year it is fire season in some part of Australia [32]. The climate in the tropical north is primarily determined by the annual monsoon with a wet season from November to April and a dry season from April to October; most of the fires occur during the latter [18]. In central parts of Australia, the fire season occurs in spring and summer, whereas in the southern parts of Australia the peak fire season generally occurs in the summer months (Dec-Feb) extending into autumn [5].

As noted by [32], the vegetation, or fuels, vary across Australia and can be broadly classified as grassland (75% cover of Australia), Forest (22% cover) and Mallee-Heath (2% cover). Williams et al. [33] discuss how climate and fuels interact to produce fire regimes in Australia. In northern and central Australia, grasslands dominate, and the fire regime is fuel driven. This means the level of fire activity is limited by the amount of fuel available, positively related to antecedent rainfall totals in the first order. For southern and eastern parts of Australia, many fires occur in forested regions (along with many grass fires). In these regions, copious fuel is present but needs extended periods of dry conditions or drought to dry out in order to burn. Therefore, the preceding conditions play a significant role in the flammability of the fuels, and fire activity in these areas can be considered broadly weather driven.

These differences in fuel and weather across Australia mean that the relevance and use of FFDI to explain fire behaviour varies across Australia. FFDI does not capture every one of the four switches and is tuned for forest conditions. Hence, it is better suited for eastern and southern parts of Australia rather than fuel driven regimes of the north and grasslands elsewhere. However, we are primarily interested in its use as a general proxy for the climate of fire weather, not the specific fire behaviour. Hot, dry and windy conditions are constant factors for dangerous fire weather across all regions and FFDI captures these well. So, while use of FFDI will introduce some limitations as a standard across Australia, it is suitable for the purposes of this study.

### Climate index data

As noted in the Introduction, we consider the variability in fire weather in relation to the ENSO, IOD and SAM. Each mode of climate variability is characterised by an index whose values reflect the state of that mode. Typically, these modes are characterised by multiple indices, with the different indices representing separate approaches and/or different datasets; these often yield subtle differences in the timing and strength of a given mode. We have considered multiple indices in this work (not shown here). The different indices do yield slightly different results; however, these differences are not large and do not affect the broad conclusions presented in this work.

The climate indices chosen for this work are summarized in Table 2. For ENSO, we select the NINO3.4 SST index (N34) based on the NOAA ERSST v5 dataset [34], an average of SST over a pre-defined region of central Pacific. The warmest (coolest) values of N34 are indicative of El Niño (La Niña) conditions. To characterize IOD, the Dipole Mode Index (DMI) [12] is chosen. This index represents the difference between SST anomalies in the western and eastern Indian Ocean. Positive values indicate anomalously warm conditions in the west and anomalously cool conditions in the east; negative conditions are the opposite. The underlying SST data come from the HadISST dataset [35]. The SAM index used here is from [36]. It is derived from standardized zonal average mean sea level pressure differences between 40°S and 65°S from 12 surface observation stations. Positive values indicate relatively higher pressure in the mid-latitudes, along with a general poleward displacement of the eddy-driven jet stream and associated storm tracks.

**Table 2:** Climate driver index name and data source.

Climate Variable	Index	Source
ENSO	NINO3.4 SST	http://www.cpc.ncep.noaa.gov/data/indices/
IOD	Dipole Mode Index	https://www.esrl.noaa.gov/psd/gcos_wgsp/Timeseries/DMI/
SAM	Meridional pressure gradient	http://www.nerc-bas.ac.uk/icd/gjma/sam.html

### Analytical methods

**Fig 2:**
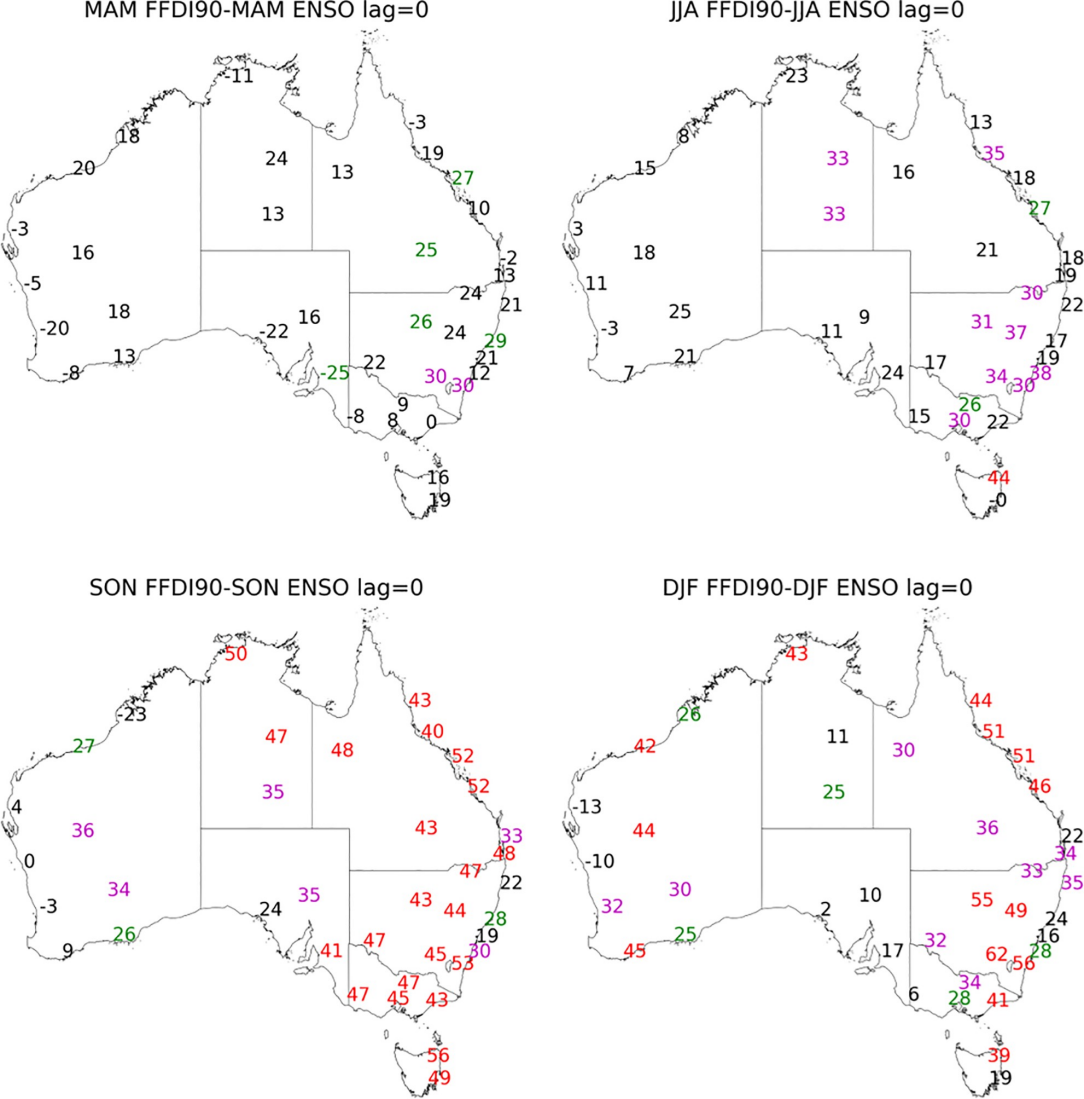
Correlation coefficient values multiplied by 100 calculated for seasonal 90^th^ percentile FFDI and seasonal N34 (a) MAM, (b) JJA, (c) SON and (d) DJF (1972–2017). Significance greater than 99% in red, 95% in magenta and 90% green.

**Fig 3:**
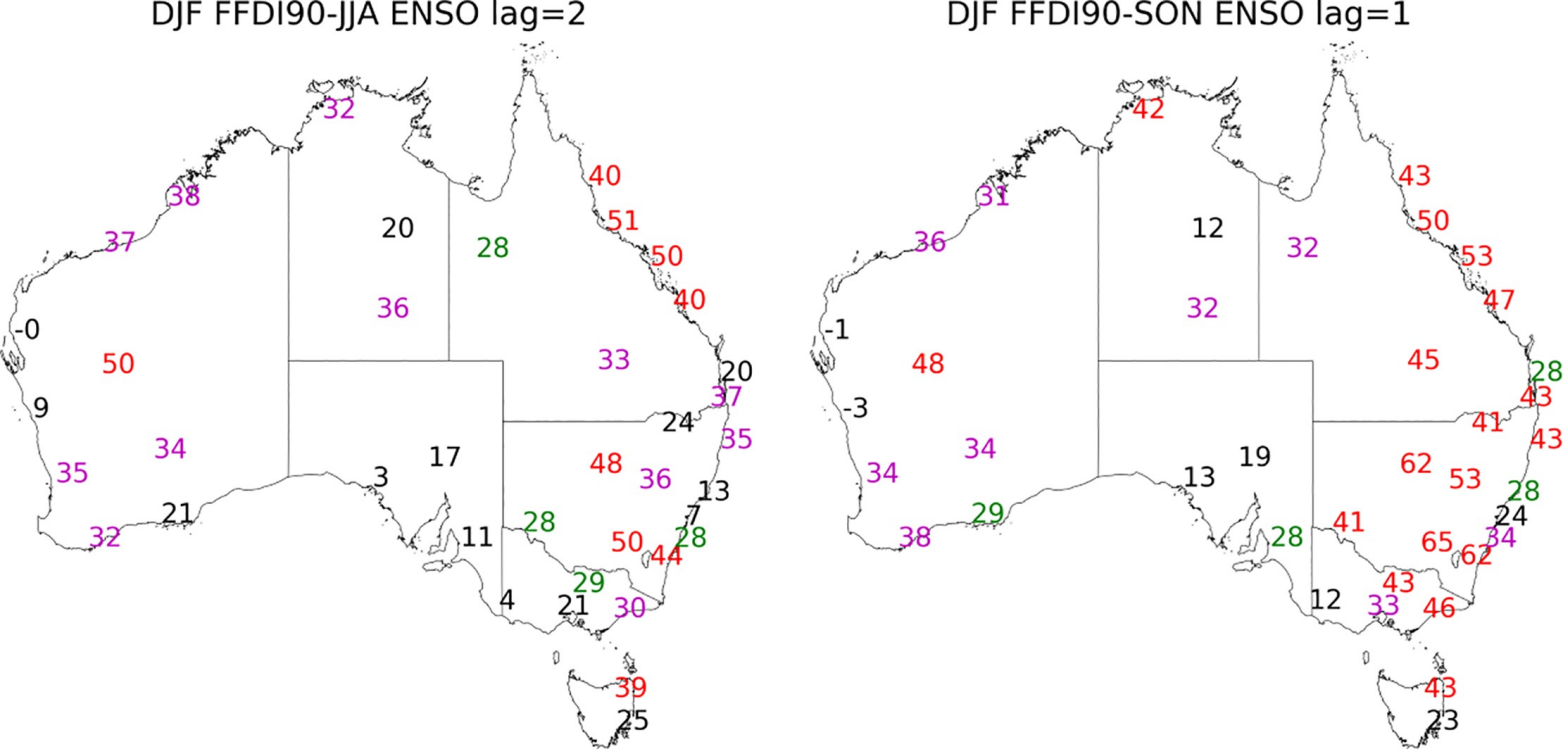
Correlation coefficient values multiplied by 100 calculated for DJF 90^th^ percentile FFDI and the preceding a. SON N34 (one-season lag) b. JJA N34 (two-season lag). Significance greater than 99% in red, 95% in magenta and 90% green.

**Fig 4:**
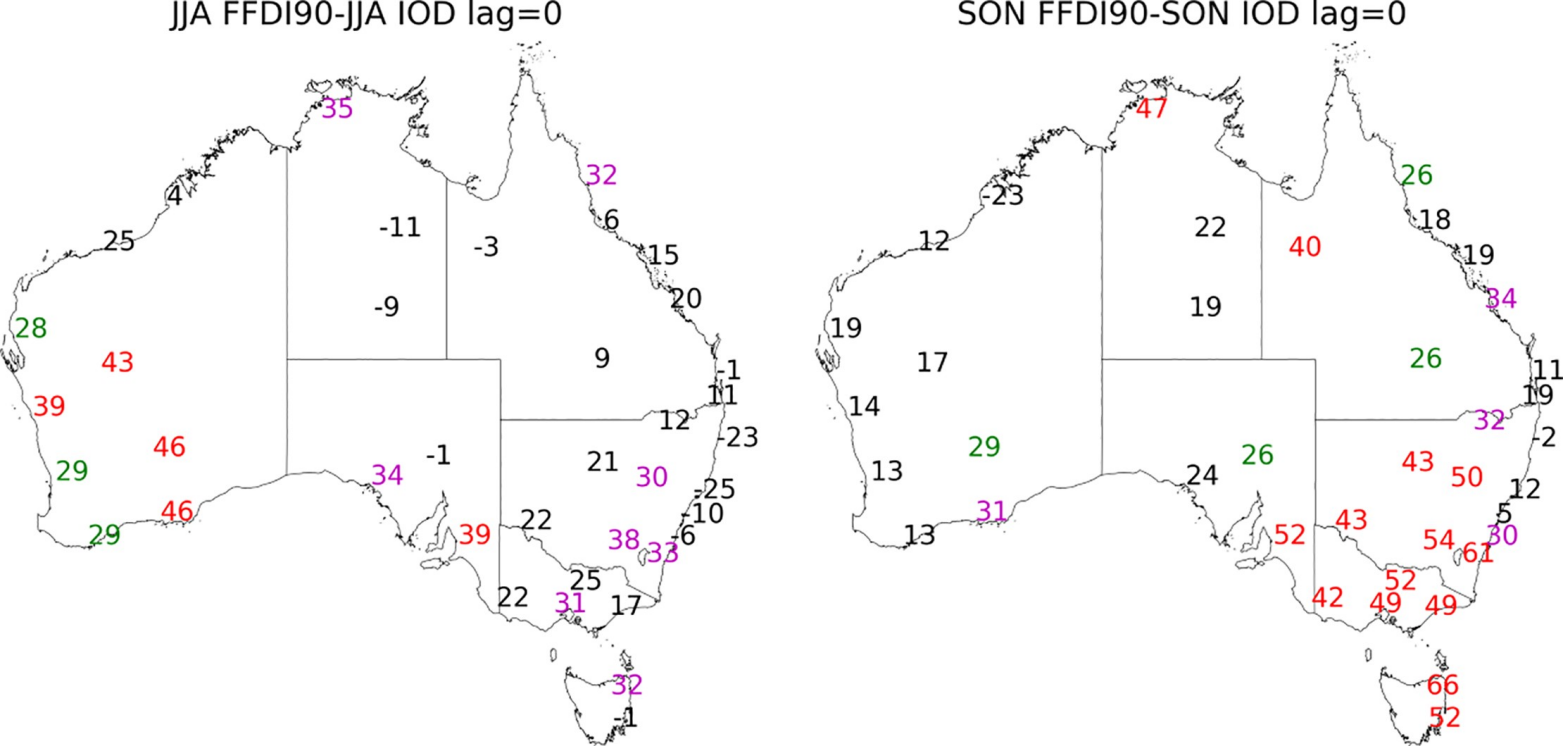
Correlation coefficient values multiplied by 100 calculated for seasonal 90^th^ percentile FFDI and seasonal DMI (a) JJA, (b) SON (1972–2017). Significance greater than 99% in red, 95% in magenta and 90% green.

**Fig 5:**
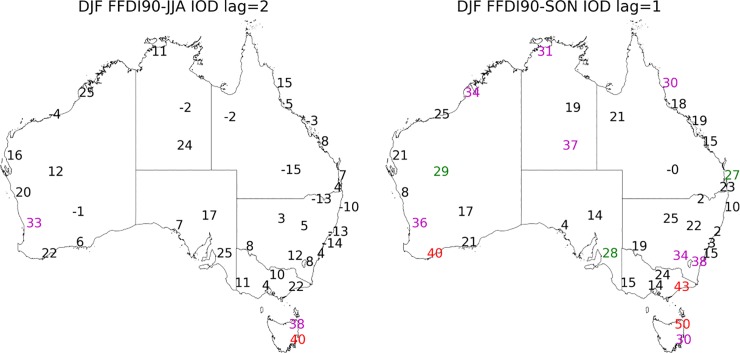
Correlation coefficient values multiplied by 100 calculated for DJF 90^th^ percentile FFDI and the preceding a. SON DMI (one season lag), b. JJA DMI (2 season lag) (1972–2017). Significance greater than 99% in red, 95% in magenta and 90% green.

**Fig 6:**
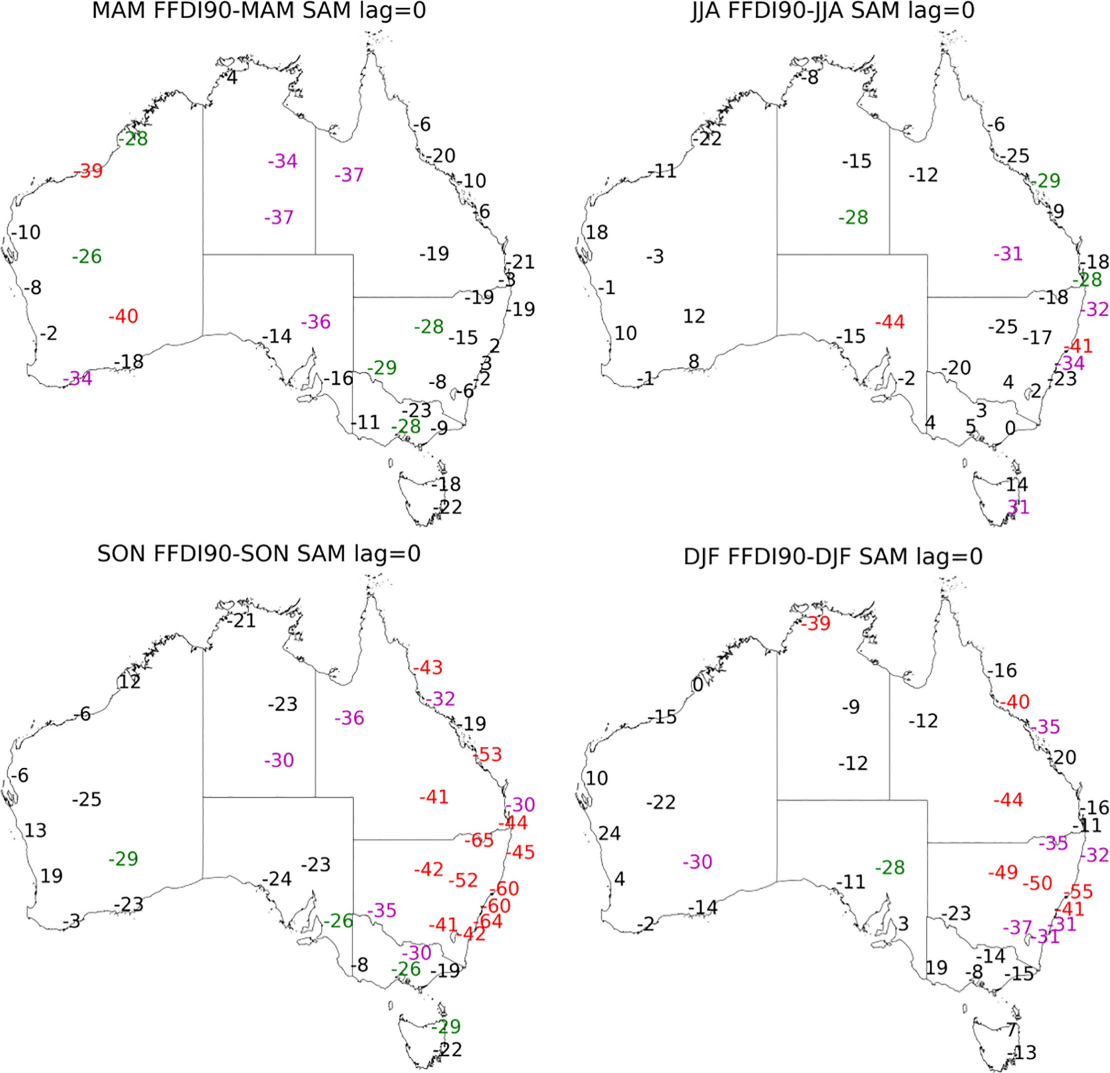
Correlation coefficient values multiplied by 100 calculated for seasonal 90^th^ percentile FFDI and seasonal SAM (a) MAM, (b) JJA, (c) SON and (d) DJF (1972–2017). Significance greater than 99% in red, 95% in magenta and 90% green.

**Fig 7:**
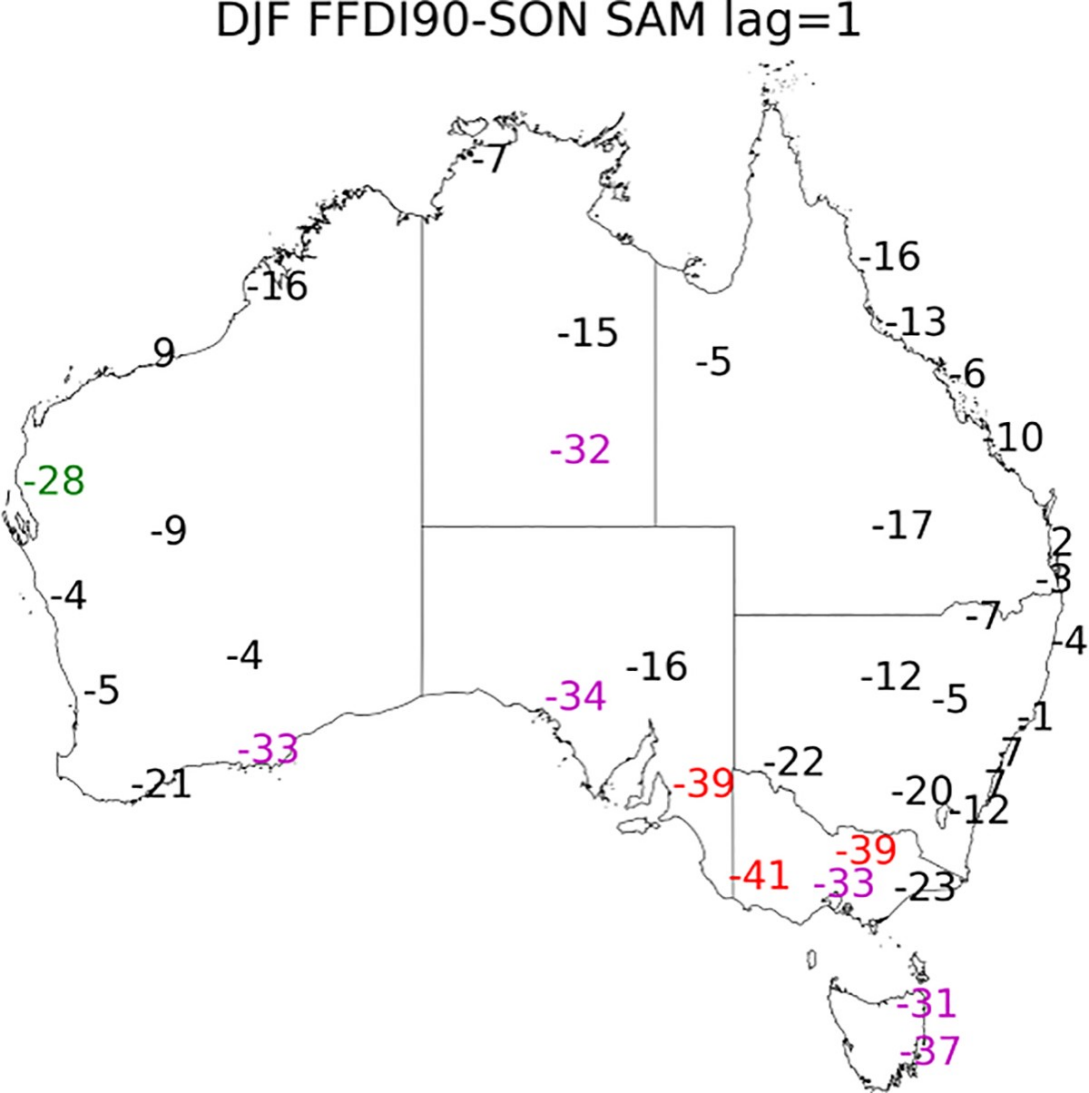
Correlation coefficient values multiplied by 100 calculated for DJF 90^th^ percentile FFDI and the preceding SAM (a) SON (one season lag), (b) JJA (two season lag) (1972–2017). Significance greater than 99% in red, 95% in magenta and 90% green.

This study primarily uses statistical methods to arrive at its conclusions. The main approach is the use of full and partial correlation analysis to examine the dependence between climate drivers and seasonal FFDI90. Partial correlation analysis is used to isolate the effects of the chosen climate index by the linear removal of the effects of other variables that are also correlated. Both concurrent and lagged relationships are considered in the correlations, with all variables linearly de-trended to capture only the interannual relationships, rather than the effects of congruent trends. Statistical significance is generally assumed at the 95% confidence level, although the additional levels of 90 and 99% are demarcated to help interpret the Figures and the strength of the relationships. Confidence levels are computed using number of samples (*N)-2* degrees of freedom for full analysis and ranging between *N-3* or *N-4* degrees of freedom for partial analyses (*N-4* if IOD is included in the analyses otherwise *N-3*). Figs 2–7 summarise the results of the full correlation analyses between seasonal fire weather and the various climate drivers. Results are presented on a map by the correlation coefficient (*r*) multiplied by 100 plotted at the location of each station (Fig 1). Scores are colour-coded to highlight their statistical significance, with correlations at or exceeding 99% significance level shown in red, the 95% level in magenta and the 90% level in green. All other values are in black. Some scores are displaced from their exact location to improve clarity (Darwin, Laverton, Melbourne Airport and Wagga Wagga). In analysing the results, we generally focus on spatially coherent groupings where the 95% significance levels have been reached. We present selected results showing the concurrent relationships (no lag) and then some one- or two-season lags (i.e. the seasonal climate index *leads* the seasonal FFDI90 by the lag amount). The analyses were for all combinations of variables and lead times, with only the more meaningful results presented here; all other results are available in the Supporting Information (S1–S18 Figs).

Composite analysis is used to further explore the relative importance of the different climate drivers. This was achieved by creating composite FFDI90 values based on the extreme high and low phases of each mode of climate variability, defined as the average of FFDI90 in the top and bottom 9 (approximately quintiles) values of each climate index for a given season. Statistically significant differences in FFDI90 between the high and low phases of the index are assessed with a Student t-test using the null hypothesis that the means between the periods are equal. The focus for these composites and the associated Figures is on the capital cities and other selected sites for SON and DJF, the active fire season for southern Australia.

Finally, linear trends are estimated using ordinary least squares regression of annual and seasonal means of 90^th^ percentile regressed against time (Julian Day). For the 'all-stations' average trend, annual FFDI90 values are standardized (by removing the mean and dividing by the standard deviation) before computing the averages; this removes potential artefacts resulting from combining data with different mean and variance. Significance of the trend is assumed when its value exceeds the 2-sigma confidence interval. When calculating the confidence interval, the autocorrelation of residuals is considered, resulting in a reduced number of degrees of freedom [37]. Trends can be difficult to interpret, particularly with a short time series. Hence, the results are valid only over the time period considered and the choice of start and/or stop times to compute the trend can strongly influence the result. Clarke et al. [4] investigated this latter sensitivity of the trend to an earlier version of the same dataset used here (7 fewer years of data) and suggested that this influence was minimal. This has been tested for this dataset (not shown here), and while the trend is sensitive to changes in start and end point, the results are still statistically significant.

## Results

### Full correlations

#### El Niño Southern Oscillation (ENSO)

Across Australia, the correlations between FFDI90 and N34 SST are positive (Fig 2). The positive relationship here indicates that FFDI90 is higher when SSTs are higher (which also occurs during the El Niño phase of ENSO), indicating enhanced fire weather during these seasons. The strength of the correlation between N34 and FFDI90 varies with season (Fig 2). During all seasons, the correlations tend to be stronger in eastern Australia. During MAM, the correlations between N34 and FFDI90 are generally weak. Some marginal correlations are identified in NSW during this season. These results are not surprising, as this season is generally the end of an old ENSO cycle or the beginning of a new one [11]. During JJA, the N34/FFDI90 relationship is weak, but stronger relationships are beginning to develop in central parts of NT, NSW and VIC. While relationships are becoming significant, the amplitude remains small. The seasonal cycle still dominates, and fire danger remains low in VIC and SA even in El Niño years during this season (JJA).

N34 has the strongest influence on FFDI90 during spring (SON). During this season, the relationship is strong and widespread with all states and territories indicating statistically significant correlations. Exceptions are seen in coastal NSW, coastal WA and Ceduna in SA. In DJF, the significant correlations are still widespread but have a smaller spatial footprint. The strength of the relationship has slightly increased in NSW/ACT and WA, while decreasing in QLD. In much of SA, TAS and VIC, there is no significant relationship.

When lag seasons are considered we find the DJF FFDI90 is significantly related to the SON N34 (one-season lag) for the same stations as found to be significant with the DJF N34 (no lag) and in most cases this relationship is stronger than with the concurrent season’s N34 values (Fig 3). When a two-season lag is considered, DJF FFDI90 with JJA N34, a similar relationship is evident as with concurrent and one-season lag for DJF FFDI90, but the strength of relationship is slightly reduced. The ENSO cycle has a pronounced multi-seasonal effect on fire weather across Australia. All other season and lag combinations between N34 and FFDI90 are available in S1–S3 Figs.

#### Indian Ocean Dipole (IOD)

During the usual active seasons of the IOD (i.e. JJA, SON), a mostly positive relationship exists between DMI and FFDI90 particularly in the west and south of Australia. In JJA, the spatial range of the correlations between the DMI and FFDI90 is more westward, with all stations in WA and the coastal stations of SA showing significant relationships. There are also some significant relationships in Victoria and central NSW/ACT (Fig 4). In SON the relationship between DMI and FFDI90 is no longer significant for most stations in WA. However, most stations across the south east of Australia are now significantly related.

For the DJF FFDI90 there is a one-season (SON DMI) and two-season (JJA DMI) lag relationship evident with DMI for both Tasmanian stations and also for Perth (WA) (Fig 5). The one season lag relationship is also evident in the very south east of Victoria and NSW/ACT. There are no other stations with a statistically significant two-season lag relationship but there are one-season (SON DMI) relationships with DJF FFDI for Albany and Broome (WA), Sale (Victoria), Charleville (QLD), Wagga (NSW), Canberra (ACT) along with Alice Springs and Darwin (NT) (Fig 5). All other season and lag combinations between DMI and FFDI90 are available in S4 Fig.

#### Southern Annular Mode (SAM)

Across much of Australia, the correlations between SAM and the FFDI90 are (generally) negative, but only show significant correlations during some seasons and in some areas (Fig 6). There are a few seasons and stations where the correlation is positive as noted below.

During MAM, SAM is significantly related to FFDI90 for mostly inland stations across Australia (Fig 6). In JJA, the relationship with the inland sites is no longer significant for most stations, with some of the NSW coastal stations have a significant negative correlation during this season. There is also a significant positive relationship in Hobart (TAS). In SON, the relationship between SAM and FFDI90 is significant for almost all stations across NSW/ACT and QLD. In DJF the relationship between FFDI90 and SAM is still significant for all stations in NSW/ACT but is slightly reduced in magnitude. In other eastern states, the relationship between FFDI90 and SAM is no longer statistically significant for most stations.

For the DJF FFDI90 the one-season lag (SON SAM), most stations in SA, VIC and TAS (Fig 7) have a significant negative correlation. All other season and lag combinations between SAM and FFDI90 are available in S5–S7 Figs.

### Composite analysis

**Fig 8:**
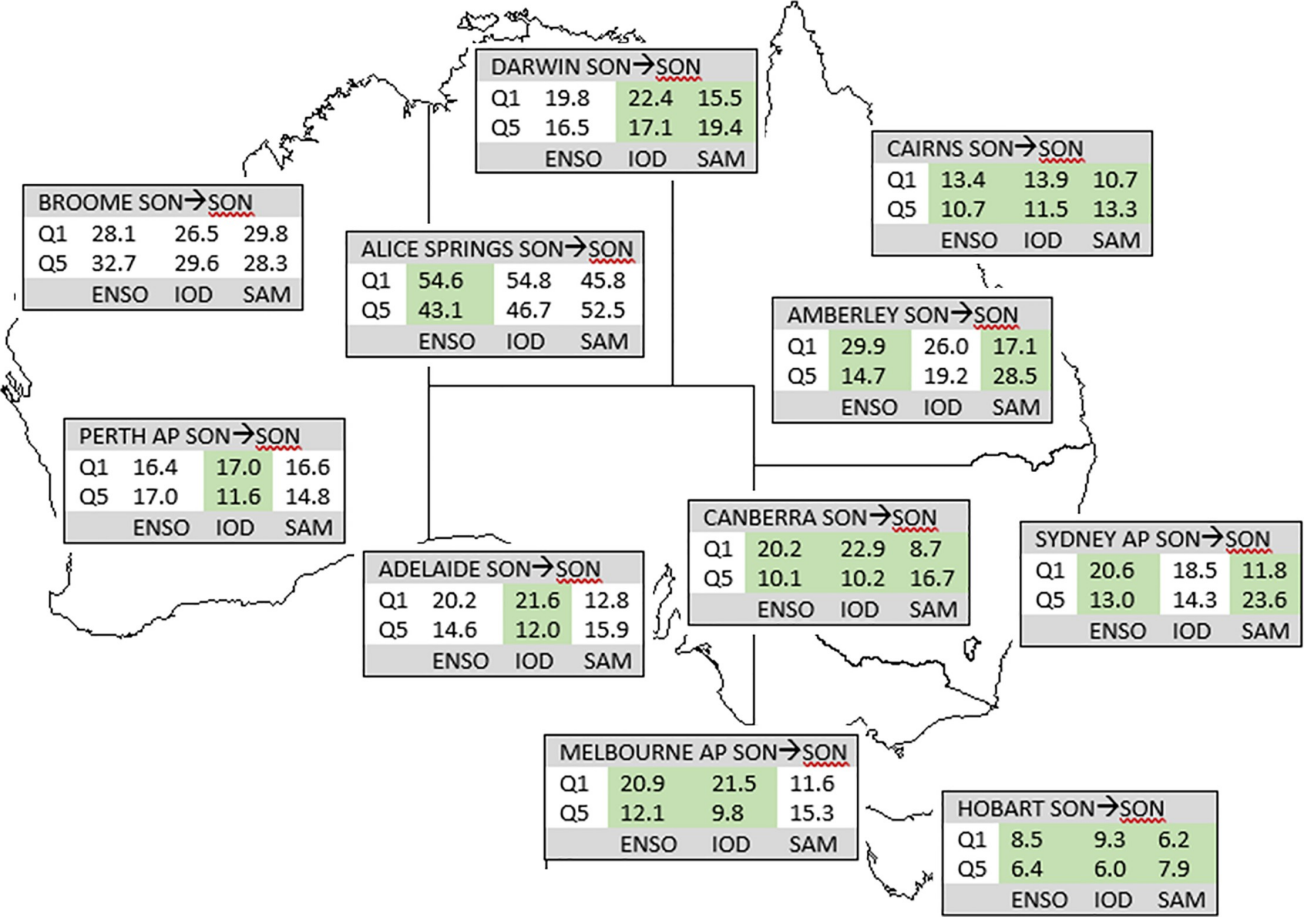
**Mean FFDI90 during SON values for top (Q1) and bottom (Q5) quintile climate states (ENSO, IOD, SAM) during SON for capital cities.** Shaded columns indicate a statistically significant difference between the two quintiles for that climate driver.

**Fig 9:**
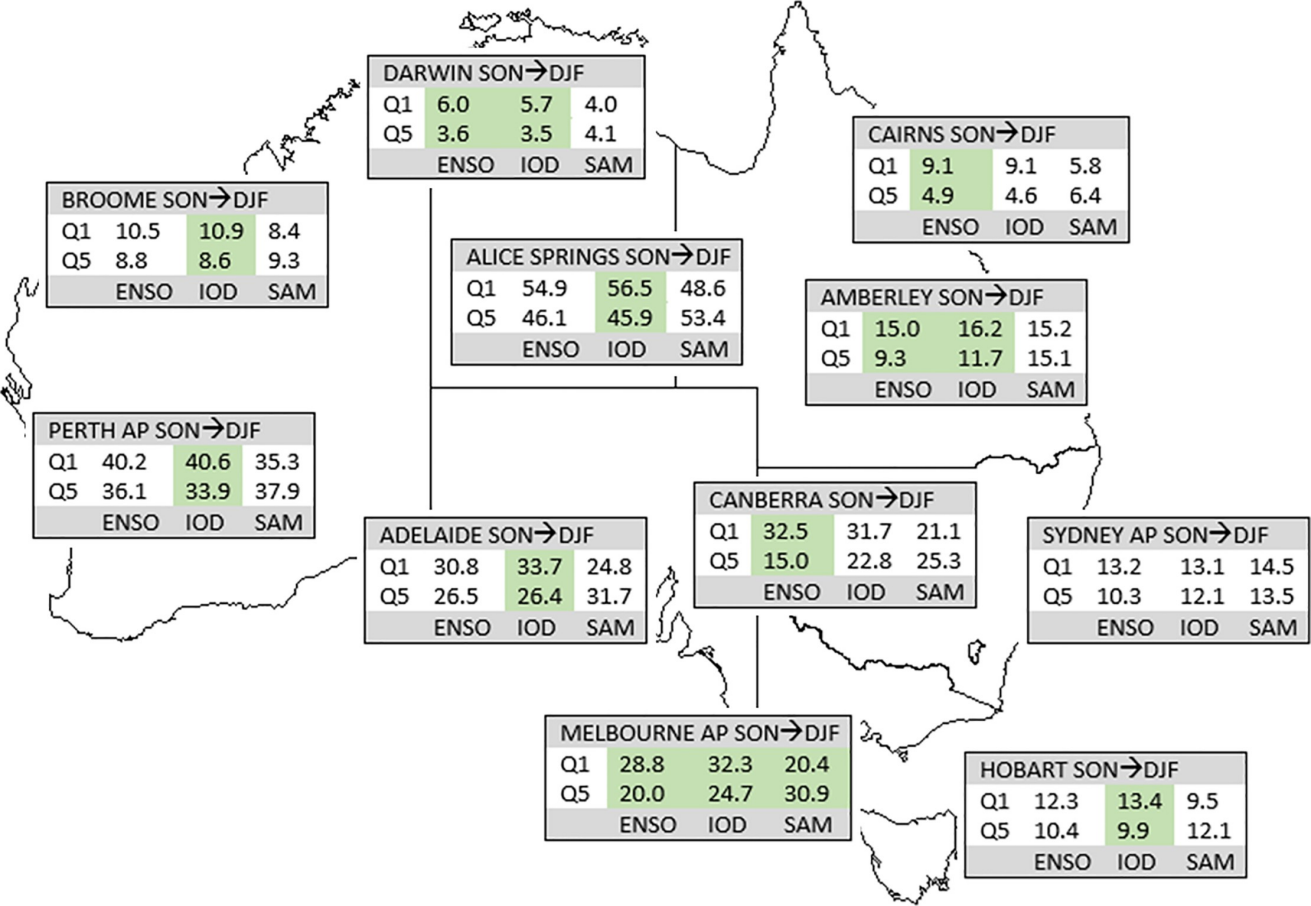
**Mean FFDI90 during SON values for top (Q1) and bottom (Q5) quintile climate states (ENSO, IOD, SAM) during DJF for capital cities (one season lag).** Shaded columns indicate a statistically significant difference between the two quintiles for that climate driver.

We further explore the relative importance of the different climate drivers by creating composite FFDI90 values based on the extreme high and low phases of each mode of climate variability. Fig 8 presents average FFDI90 in SON based on the sorted SON climate modes (in S2 Table); Fig 9 presents average FFDI90 values for DJF based on SON rankings, reflecting a one-season lag. While there is considerable overlap in the years used in each composite, especially between ENSO and IOD, the years used are not identical and these differences allow insight into the relative importance of the different climate modes.

For the zero-lag case (Fig 8), there are generally significant differences in FFDI90 between the highest N34 SSTs and the lowest N34 SSTs; even where not significant, the differences are often large (e.g. Adelaide); Perth and Broome are exceptions, consistent with the lack of significant correlations noted earlier. For IOD, the positive phase generally has larger average FFDI90 values compared to the negative phase; the relationships are not significant at Alice Spring, Amberley or Sydney, and Broome shows the opposite tendency. At the stations where the difference is significant, the average FFDI90 values during positive IOD are larger than those for the highest N34 SSTs. This suggests a mutual effect where positive IOD in conjunction with higher N34 SSTs, creates a worse fire weather situation than either alone. With SAM, a statistically significant difference is observed between the negative and positive phases at most stations. The negative phase mostly has higher FFDI90, with the WA stations the exception here. At Sydney, the negative SAM average FFDI90 is greater than the higher N34 SSTs, with little overlap between the sets of years. Regardless of statistical significance, these differences generally align with the sense of the correlations indicated here; FFDI90 is higher during higher N34 SSTs and positive IOD and negative SAM at most stations.

For the one-season-lag case (i.e. DJF FFDI90 stratified by SON climate modes, Fig 9), ENSO and IOD produce the strongest differences. In the western and southern portions of the country, IOD produces the largest differences in FFDI90 between positive and negative phases. Further, at many stations the overall highest values are often found during years with positive IOD. ENSO produces similar differences at most stations, but these are only statistically significant at a few stations. A lagged SAM effect is apparent at many stations in the southeast, but only significant at Melbourne.

We have examined the effects of JJA climate variables lagged into DJF (two seasons; shown in S13 Fig). In this case, there are strong differences associated with ENSO across the southern and eastern parts of the country (Sydney excluded). At Adelaide, Perth, Alice Springs and Hobart, significant differences are seen with IOD. At Sydney, positive SAM in JJA results in higher DJF FFDI90 compared to negative SAM, reflecting the complexity of the SAM-rainfall relationship in Australia.

### Partial correlations

#### Interdependence of predictors seasonally

The seasonal interdependence between the climate drivers emphasises the need for a partial correlation analysis so that the individual effect of each driver on FFDI can be estimated. Table 3 presents the seasonal correlations between the different climate factors. Positive IOD and El Niño are often concurrent, with roughly 70% of positive IOD events occurring simultaneously with El Nino [38]. This is reflected in the statistically significant correlation values between DMI and N34 during the relevant seasons (JJA, SON). The relationship is particularly pronounced during SON. These results are similar to those reported by [23] for the 1979–2008 period.

**Table 3:** Correlation coefficients between seasonal climate drivers NINO3.4, DMI and SAM for the period December 1972 to May 2017. Results for DMI only include JJA and SON when IOD is usually formed. Bold indicates statistically significant p<0.05.

	Climate Driver	N34	DMI
MAM (n = 45)	DMI		
	SAM	-0.09	-0.11
JJA (n = 44)	DMI	**0.33**	
	SAM	0.12	0.20
SON (n = 44)	DMI	**0.68**	
	SAM	-0.16	-0.24
DJF (n = 45)	DMI		
	SAM	-0.23	

The SAM is not significantly correlated with the other climate indices examined here (Table 3). While this suggests little effect, previous studies have indicated that negative SAM is dominant during El Niño events and positive SAM preferentially occurs during La Niña events [39,40] during austral spring and summer. However, this relationship is dependent on the amplitude of ENSO; during weaker or neutral cases, the relationship disappears. This is consistent with the weak negative correlations (|*r*| ~0.15–0.25) during SON and DJF. Significant interactions between SAM, ENSO and Australian rainfall, particularly during SON and DJF, have also been documented [41,42].

#### Concurrent seasons

**Fig 10:**
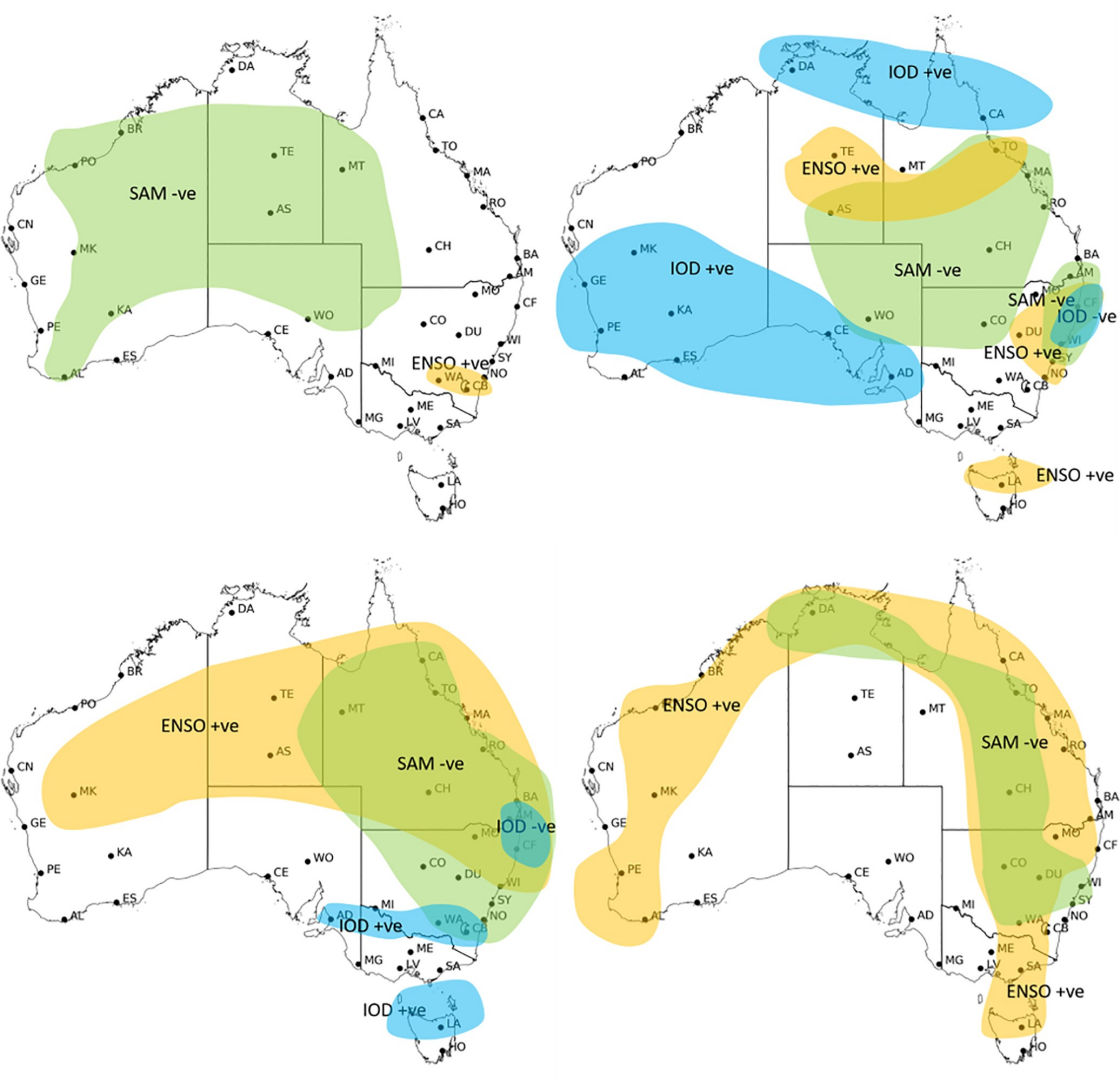
Subjectively produced regions in a) autumn (March to May), b) winter (June to August), c) spring (September to November) and d) summer (December to February) with statistically significant partial correlation (p>0.05) between 90^th^ percentile FFDI and climate drivers at 39 stations grouped in spatially coherent patterns 1972/73 to 2016/17. ENSO (N34) is orange, SAM (SAM) is green and IOD (DMI) is blue.

**Fig 11:**
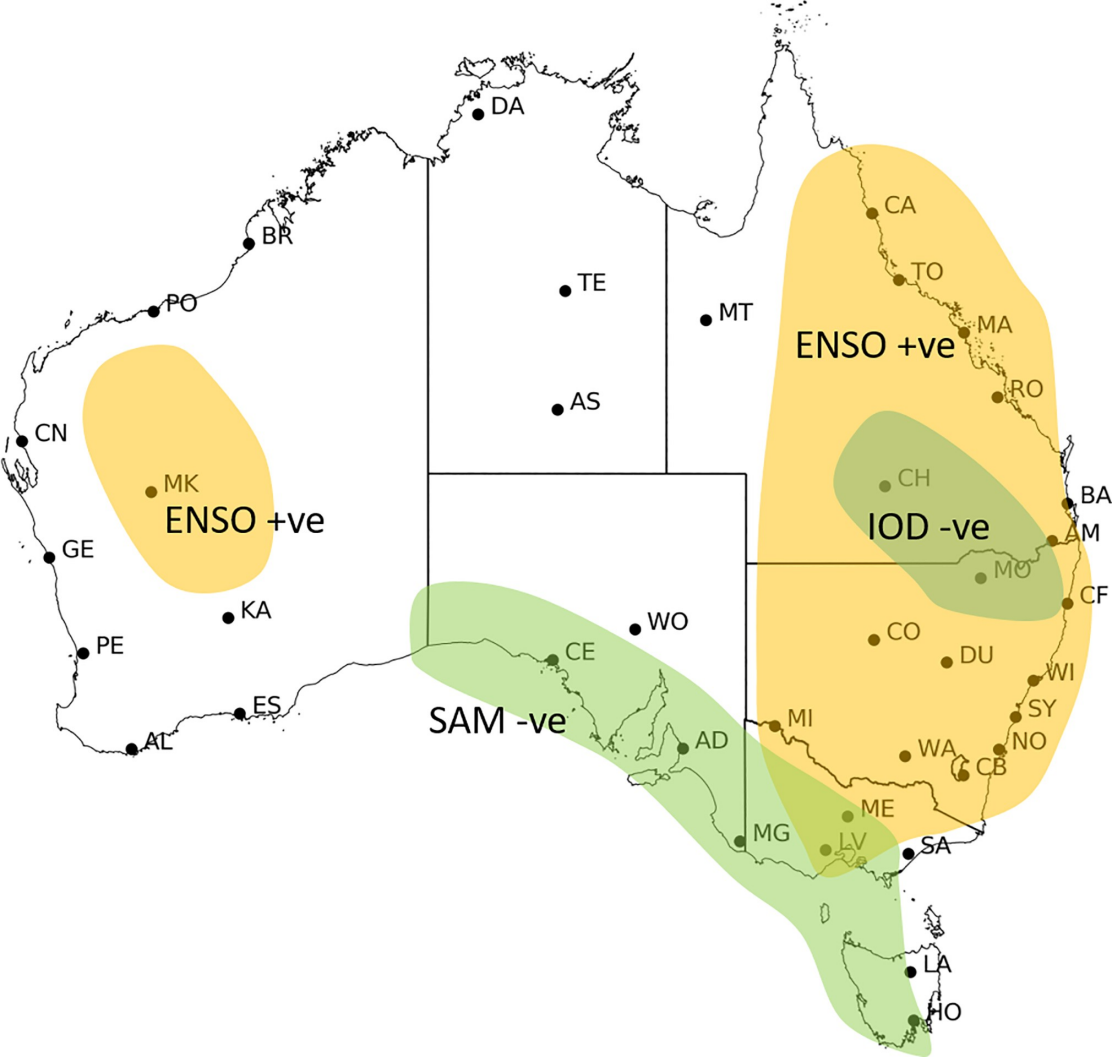
Subjectively produced regions with one season lag relationship between summer 90^th^ percentile FFDI and spring climate drivers with statistically significant partial correlation (p>0.05) at 39 stations grouped in spatially coherent patterns 1972/73 to 2016/17. ENSO (N34) is orange, SAM (SAM) is green and IOD (DMI) is blue.

The interactions between climate drivers along with their combined effects on weather are complex. However, it is still useful to have an approximate indication of their relative influences in different places and seasons [9]. Calculating partial correlations reveals the relative influences of the different drivers independent of the combined effects in each location and season, along with lag seasons and in some cases, there is more than one driver dominating. The broad regions influenced by the particular region have been grouped together for concurrent seasons in Fig 10 and Fig 11 for lag seasons. These groupings should only be interpreted as qualitative, with maps of the correlation coefficients available in Supporting Information (S8–S18 Figs).

During MAM, SAM dominates the inland stations of NT, SA WA, while N34 has a significant relationship with two stations in the south east of NSW/ACT (Fig 10A). IOD is not included in these analyses because the phase does not generally exist in that season.

During JJA, N34 dominates in central NT, northern TAS and most of NSW (Fig 10B). SAM dominates across NSW, inland NT and QLD and some coastal QLD stations. IOD dominates in southern WA and SA and also in northern NT and QLD. The result for the full correlations between JJA N34 and JJA FFDI90 is strong and significant whereas this relationship is reduced when the effects of the other climate drivers are removed. In most regions, the relationship between IOD and FFDI is positive. However, in parts of NSW there is a negative relationship between FFDI and IOD in the partial correlations. This indicates that the other climate drivers (mostly ENSO) play a major role in controlling the relationship between IOD and FFDI. This finding is also supported by previous work that shows enhanced rainfall during a positive IOD (see[43]).

During SON, N34 has a positive relationship with FFDI90, dominating the northern and central east coast across to inland western Australia. SAM has a negative relationship with FFDI90 and is also a dominant driver across QLD but extends to the whole of NSW. IOD has a weak negative relationship in two coastal stations in the east, although the ENSO effect is stronger. IOD has a positive relationship with FFDI90 and dominant in TAS across to Adelaide in SA (Fig 10C). In VIC, IOD and N34 are separately significantly correlated with FFDI90 but without the combined effect neither of the variables are statistically significant.

In DJF, N34 is the dominant driver of fire weather for most of the coastal stations; exceptions include some WA stations, some NSW stations and all of the SA stations (Fig 10D). N34 also dominates the inland stations in QLD and WA. SAM is the dominant driver of FFDI90 for the NSW stations extending into a central QLD.

#### Lag relationships

When the one-season lag effect of SON climate drivers on DJF FFDI90 is considered (Fig 11), N34 dominates most of NSW, QLD, VIC and central WA, while SAM is the dominant driver of fire weather in the south extending from WA through to VIC, SA and TAS. For the WA stations, the full correlation results with DMI and N34 are significantly correlated separately for many stations. However, when considered independently the statistically significant correlations largely disappear, with only Kalgoorlie and Meekatharra maintaining weak relationships with N34. This suggests that the two variables occurring simultaneously is particularly important. Further, in parts of NSW the relationship between the DMI and FFDI90 is not significant however when the effects of the other climate drivers are removed the relationship of DMI in this region becomes negative.

When a two-season lag is considered for DJF FFDI90 (with JJA climate drivers) the dominant driver of fire weather is N34 in NSW/ACT, NT and QLD and this has been strengthened following the removal of the effects of SAM and DMI. N34 also still dominates in WA but this is less widespread for partial correlations (refer to Figs of partial correlations in S8–S18 Figs).

For the one-season lag effect on SON FFDI90 from the JJA climate drivers, N34 still dominates in NSW/ACT for most stations with a significant relationship with IOD still evident for Cobar, Coffs Harbour and Moree. N34 also dominates in NT and also in QLD although IOD has been strengthened for some stations. The effect of N34 in VIC has again been reduced by the removal of the effect of the other drivers (refer to S8–S18 Figs).

### Trends and variability

#### Fire weather

**Fig 12:**
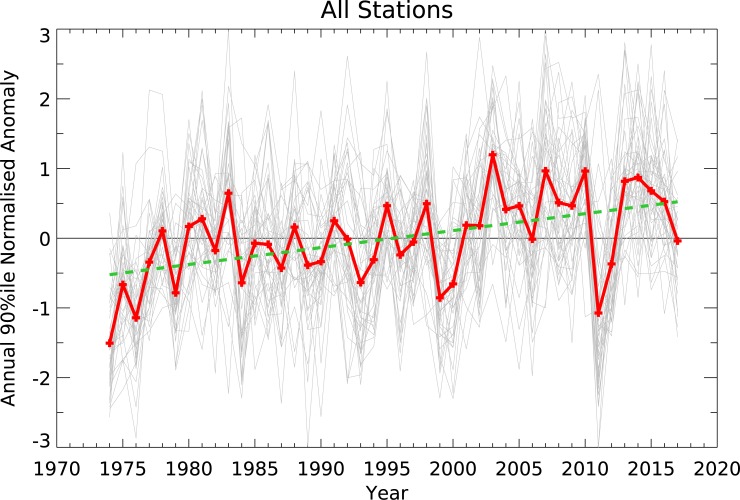
Time series of 90^th^ percentile FFDI annual anomaly (July-June) at each station (1973–2017). The thick line indicates the multi-station mean. The thick dotted line indicates the linear trend.

The time series of annual FFDI90 are characterised by a significant amount of interannual variability and a general upward trend, both at individual stations and in the all-station mean (Fig 12). There is a considerable degree of coherence between the stations in these signals. Judging from the fire weather time series, the fire seasons of 1973–74 and 2010–11 are the two mildest seasons over the past several decades. These periods are the wettest on record for Australia and coincid with moderate to strong La Niña events (see [44]). Since the early 2000s, the multi-station mean shows that most years have had higher-than-average fire weather, with the most severe season being 2002–03. This season coincided with major rainfall deficiencies and exceptionally warm conditions across most of Australia, and a weak to moderate El Niño event (see [45]). Individually all stations have a positive annual trend for FFDI90 except for Brisbane Airport. This negative annual trend occurs due to negative trends in FFDI90 in both summer and autumn and is not statistically significant, also found by [4]. For the all-station average, the standardized trend is 0.24±0.13 per decade, equivalent to 0.96±0.52 points per decade using the average value of standard deviation (4.01) across all stations. The use of the standardized trend results in a decrease of 7% in trend magnitude and a decrease of 15% in the size of the confidence intervals compared to the non-standardized values. In either case, these results represent a continuation of the trends (in the same variables) identified by [4]; the overall annual trend values have lessened and this is most likely due to the very mild 2010–11 period, but the general upward trend remains statistically significant.

**Fig 13:**
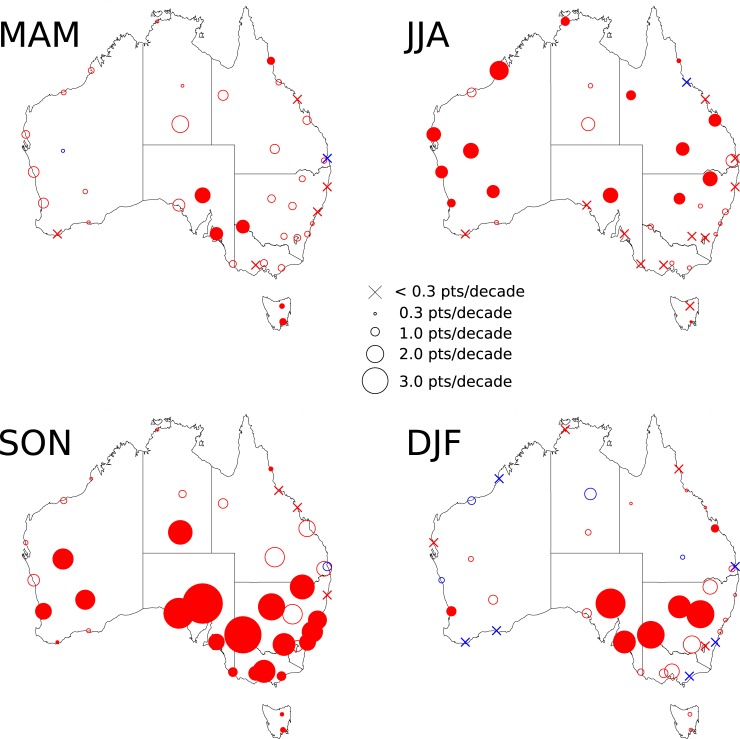
Map of trends in seasonal 90^th^ percentile FFDI. Marker size is proportional to the magnitude of trend. Reference sizes are shown in the legend. Filled markers represent trends that are statistically significant. Red indicates an upward trend and blue indicates a downward trend.

The trend magnitudes vary in both a spatial and seasonal sense (Fig 13 and Table 4). Trends are strongest and most widespread during the spring (SON), encompassing much of southern Australia. Further north, weaker but statistically significant trends are identified during JJA. Taken together, these trends imply an earlier start to the local fire season. Overall, the strongest changes are occurring during the earlier parts of the fire season. During DJF, strong positive trends are largely confined to a regional area of eastern SA, western NSW and northwest Victoria. Trends are larger in other parts further east, but only significant at the 90% level. During MAM, the same regions reveal statistically significant trends, but these are weaker compared to spring as was also found previously [3,4].

**Table 4:** Values of seasonal FFDI90 and FFDI50 trends from 1973–2017 (2018 for DJF). Trends (top row of each cell) in points per decade. 2^nd^ column of each cell represents the 2-sigma confidence interval. Trends significant at 95% level (greater than 2-σ CI) are in bold; values in italics are significant at ~90% level (1.65*CI).

Site	DJF FFDI90	MAM FFDI90	JJA FFDI90	SON FFDI90	ANNUAL FFDI90
Adelaide	**2.64 ± 1.43**	**1.55 ± 1.49**	0.15 ± 0.55	**1.90 ± 1.52**	**1.88 ± 0.97**
Albany	-0.20 ± 0.58	0.22 ± 0.60	0.26 ± 0.18	**0.44 ± 0.31**	0.04 ± 0.35
Alice Springs	0.67 ± 2.25	1.95 ± 3.23	*1*.*53 ± 1*.*67*	**2.84 ± 2.72**	1.40 ± 2.15
Amberley	0.71 ± 1.13	0.63 ± 1.03	*1*.*41 ± 1*.*46*	1.71 ± 2.14	1.00 ± 1.63
Brisbane	-0.26 ± 0.57	-0.19 ± 0.53	0.17 ± 0.94	-1.00 ± 1.40	**-0.56 ± 0.43**
Broome	-0.27 ± 0.61	0.69 ± 1.40	**2.22 ± 0.99**	0.34 ± 1.91	**0.99 ± 0.97**
Cairns	0.18 ± 0.79	**0.93 ± 0.55**	**0.53 ± 0.45**	**0.56 ± 0.54**	**0.59 ± 0.35**
Canberra	0.19 ± 2.28	0.79 ± 1.73	0.20 ± 0.49	1.27 ± 1.96	0.33 ± 1.67
Carnarvon	0.01 ± 0.84	0.91 ± 1.12	**1.69 ± 1.04**	0.51 ± 1.04	**0.76 ± 0.56**
Ceduna	1.08 ± 2.00	1.39 ± 2.19	0.11 ± 1.47	**3.57 ± 2.82**	*1*.*20 ± 1*.*39*
Charleville	-0.49 ± 2.44	1.08 ± 1.80	**1.56 ± 1.47**	*2*.*26 ± 2*.*34*	0.53 ± 1.44
Cobar	**2.65 ± 2.37**	0.89 ± 1.63	**1.34 ± 1.26**	**3.13 ± 2.38**	**2.42 ± 1.62**
Coffs Harbour	0.35 ± 0.50	0.26 ± 0.35	0.02 ± 0.60	0.17 ± 0.53	0.20 ± 0.39
Darwin	0.06 ± 0.49	0.36 ± 1.01	**1.05 ± 0.79**	0.36 ± 1.00	*0*.*49 ± 0*.*54*
Dubbo	**3.24 ± 1.95**	0.87 ± 1.41	0.49 ± 0.86	2.28 ± 2.70	**1.86 ± 1.33**
Esperance	-0.18 ± 0.67	0.40 ± 1.08	0.48 ± 0.63	*0*.*47 ± 0*.*51*	0.11 ± 0.37
Geraldton	-0.65 ± 1.95	1.25 ± 2.02	**1.47 ± 1.24**	1.35 ± 1.93	**1.10 ± 1.05**
Hobart	0.41 ± 0.67	**0.84 ± 0.59**	**0.32 ± 0.28**	**0.66 ± 0.47**	**0.52 ± 0.35**
Kalgoorlie	1.06 ± 1.56	0.53 ± 1.74	**1.51 ± 1.48**	**2.32 ± 2.16**	*1*.*07 ± 1*.*21*
Launceston	0.45 ± 0.94	**0.64 ± 0.53**	0.11 ± 0.15	**0.50 ± 0.45**	*0*.*47 ± 0*.*53*
Laverton	1.01 ± 1.71	0.11 ± 0.85	0.01 ± 0.47	**1.71 ± 1.33**	0.48 ± 1.01
Mackay	*0*.*31 ± 0*.*38*	**0.32 ± 0.31**	0.02 ± 0.30	0.10 ± 0.30	0.17 ± 0.25
Meekatharra	0.58 ± 1.58	-0.38 ± 1.81	**1.82 ± 1.57**	**2.44 ± 1.86**	1.05 ± 1.46
Melbourne	*1*.*72 ± 1*.*84*	0.83 ± 1.48	*0*.*49 ± 0*.*50*	**2.66 ± 1.59**	**1.20 ± 1.19**
Mildura	**3.20 ± 2.37**	**1.58 ± 1.54**	0.62 ± 0.93	**4.31 ± 2.22**	**2.63 ± 1.87**
Moree	1.67 ± 2.10	0.71 ± 1.47	**1.73 ± 1.13**	**2.88 ± 2.30**	**1.69 ± 1.41**
Mt Gambier	0.74 ± 1.87	*0*.*80 ± 0*.*94*	0.01 ± 0.14	**1.10 ± 0.87**	**0.71 ± 0.67**
Mt Isa	0.30 ± 3.01	1.17 ± 2.26	**1.14 ± 1.01**	1.11 ± 1.91	0.93 ± 1.77
Nowra	-0.01 ± 1.53	0.68 ± 0.82	0.37 ± 0.72	**1.94 ± 1.83**	**0.67 ± 0.66**
Perth	**1.17 ± 1.03**	*1*.*16 ± 1*.*21*	**0.99 ± 0.48**	**1.93 ± 0.95**	**1.09 ± 0.88**
Port Hedland	-0.91 ± 2.51	0.57 ± 1.63	1.04 ± 1.25	0.72 ± 1.66	0.53 ± 1.14
Rockhampton	**0.95 ± 0.92**	0.97 ± 1.27	**1.48 ± 1.15**	*1*.*90 ± 2*.*00*	**1.25 ± 0.68**
East Sale	-0.20 ± 1.49	*0*.*78 ± 0*.*81*	*0*.*48 ± 0*.*51*	**1.10 ± 1.04**	*0*.*61 ± 0*.*71*
Sydney	0.57 ± 0.97	0.46 ± 0.77	0.43 ± 0.82	**2.46 ± 1.70**	**0.79 ± 0.63**
Tenant Creek	-1.35 ± 2.28	0.33 ± 2.74	0.52 ± 0.96	0.85 ± 2.13	0.16 ± 1.92
Townsville	0.34 ± 0.67	*0*.*65 ± 0*.*79*	-0.11 ± 0.88	0.07 ± 0.91	0.14 ± 0.55
Wagga Wagga	2.00 ± 2.56	0.73 ± 1.74	0.19 ± 0.43	**2.63 ± 2.31**	1.36 ± 1.71
Williamtown	0.44 ± 1.24	0.12 ± 0.86	0.69 ± 1.01	**2.19 ± 1.85**	**0.75 ± 0.75**
Woomera	**3.47 ± 1.61**	**1.83 ± 1.51**	**1.78 ± 1.54**	**4.68 ± 3.06**	**3.13 ± 1.70**

#### Climate drivers

Trends for seasonal climate driver indices were also examined (Table 5). Trend in SAM is significant in DJF (p<0.05), and less significant in MAM (p<0.10), it is also relatively strong in JJA, but this is not statistically significant. Trends in N34 are not statistically significant for any seasons. For DMI, there is a less significant trend during SON. These findings are supported by existing literature that have found an increase in the positive phase of SAM [36] and there are no studies identifying a trend in ENSO or IOD so far, although an increase in the frequency of El Niño events and positive IOD events due to anthropogenic global warming has been hypothesized for the future [46,47].

**Table 5:** The trend for seasonal climate driver indices (SAM, NINO3.4 (N34) and IOD (DMI)) for 1973-2016/17. Trends in bold indicate significance p<0.05 and in italics indicate p<0.10.

	DJF	MAM	JJA	SON
SAM	**0.40 ± 0.30**	0.27 ± 0.28	0.21 ± 0.35	0.01 ± 0.36
N34	0.01 ± 0.29	0.11 ± 0.19	0.14 ± 0.17	0.13 ± 0.27
DMI	0.03 ± 0.05	**0.05 ± 0.05**	0.05 ± 0.08	0.11 ± 0.12

#### Influence of climate driver trends on fire weather trends

**Fig 14:**
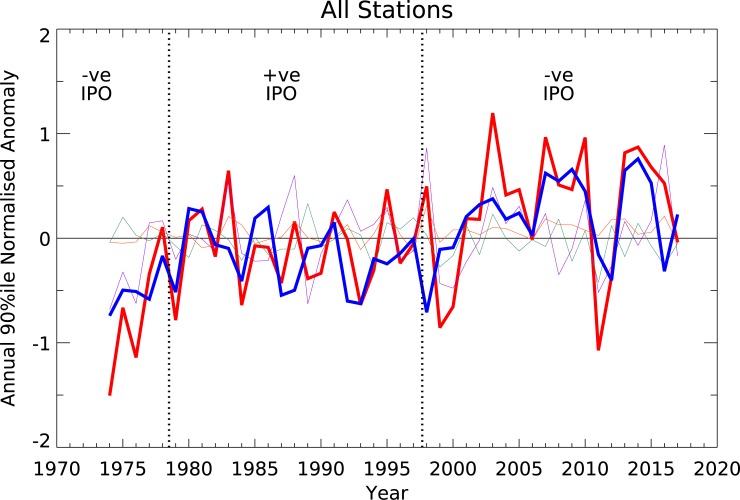
Annual all station average FFDI90 as in Fig 3 (thick red) and with effects of individual climate drivers removed (thick purple). The individual effects of N34 (cyan); IOD (orange) and SAM (green) are shown as thin lines. Vertical dotted lines indicate the transition points between the phases of the Interdecadal Pacific Oscillation during the period of record as identified by the Henley et al (2015) dataset.

To determine if the trends in climate drivers are influencing the identified fire weather trends we use partial regression to estimate the effect of each climate driver (during SON and DJF, the most influential seasons) on the 'all-stations' average annual FFDI90, reproduced as the thick red line in Fig 14. The estimated effects of each driver are shown as the thin lines in Fig 14, with the thicker purple line representing the average FFDI90 adjusted to remove the effects of the climate drivers. If the total trend were an artefact of the apparent underlying trends in the climate drivers, the purple line should lie close to the zero-anomaly line throughout; however, it does not. Trends in the climate drivers (Table 5) do explain some of the overall trends, with the total standardized trend reduced by 27% to 0.18 ± 0.11 points/decade. Trends in FFDI due to N34 and the DMI are 0.06 ± 0.09 and 0.02 ± 0.03 points/decade, respectively. SAM acts in the opposite direction with a negative trend, with a value of -0.02 ± 0.05 points/decade. A limitation of this approach is the inadequacy of the linear regression models applied here, particularly in the more extreme excursions (e.g. 1973/4 or 2010/11) from La Niña and that the influence of lag relationship is not considered. The teleconnection effects of the ENSO cycle may be non-linear [48,49] and the linear model applied here may not sufficiently capture the effects of the climate drivers, particularly at the extremes, where an asymmetrical response between El Nino and La Nina has been previously reported [50]. Regardless, the trends in climate drivers appear to fall well short of explaining the overall trend in FFDI.

## Discussion

The results presented in this study allow for a greater understanding of the drivers of Australian fire weather variability over the previous 45 years as quantified through the FFDI. This variability of fire weather is driven in part by complex interactions between major Australia climate drivers–ENSO, IOD and SAM–and superposed on an increasing background trend that is present nationally, but strongest across Southern Australia. We will discuss these drivers more completely in this section.

### Interannual variability

Not surprisingly, ENSO is the dominant driver of fire weather variability; this result is consistent with previous studies [3,22] and with its known global impact. ENSO is the most spatially widespread driver and affects most of the Australian continent to some degree, although the signal is clearly stronger in the central and eastern portions of the country. These impacts are obvious during the winter (JJA), spring (SON) and summer (DJF), and are often lagged; what happens early in the ENSO cycle persists through the seasons.

The IOD plays a significant role primarily in southern Australia. During JJA, the strongest correlations are in southern and central WA; as the season shifts to spring (SON), the strongest correlations move into south-eastern Australia. These results are similar to those of [51] who found similar seasonal variations for the IOD influence on fire weather in Australia. Generally weak correlations with IOD are also noted at several stations in northern Australia during both JJA and SON.

SAM demonstrates widespread correlations across much of the country, depending on the season. The correlation is generally negative, meaning that when SAM is persistently in its negative phase then FFDI is higher. While present across the country, this relationship is most prominent across eastern Australia; the strongest relationships centred on NSW during SON and DJF. The interior portions of the country (e.g. Mt Isa, Tennant Creek, Alice Springs, Kalgoorlie), show moderate correlations during MAM. A significant one-season lag correlation with SON SAM is also observed in parts of SA, VIC and TAS.

We suggest the different modes of variability examined here act to modulate the fire-weather climate primarily through changes in the location and timing of rainfall. An explanation for the impact may be through changes in the number of rain days and to a lesser degree, the rainfall intensity per rain day [52]. These effects on rainfall subsequently flow through to other weather variables that affect FFDI. To first order, lower-than-average rainfall can be linked to a reduction in cloudiness, increased insolation, relatively more sensible heating and less evaporation, all of which lead to higher daytime temperatures [53]. Similarly, atmospheric humidity as measured by the dewpoint on average is lower when rainfall anomalies are negative [54]. Combined with higher temperatures, this will lead to lower average values of relative humidity. Both higher temperatures and lower rainfall also increase the KBDI and drought factor [28]. These factors are all directly related to an increasing FFDI.

Spatially, the impacts of each climate mode on rainfall in Australia compare well with the correlations of those modes with 90^th^ percentile FFDI. The strongest rainfall impacts of ENSO are primarily in eastern Australia (see [9]). For IOD, the rainfall impacts are particularly strong in central Australia, VIC and TAS (see [9]), but also extend to other regions. For SAM, the rainfall response is complex, with higher-than-normal rainfall during the positive phase during SON across much of NSW, while in other seasons (e.g. JJA) the positive phase brings dry anomalies to much of southern Australia [8]. However, in this study we did not see the effect of the positive phase of SAM resulting in a statistically significant positive relationship with FFDI, with the exception of Hobart in JJA.

Temporally, ENSO is the most persistent of these phenomena. Significant SST anomalies from ENSO generally arise in late-autumn and winter, strengthen into the spring and fully mature in summer, after which they generally rapidly decay [11]. On average, the IOD has a shorter cycle, typically developing in winter (JJA), peaking in spring and rapidly decaying during November and December [12]. However, the IOD is not as persistent over the course of a given year and events can often terminate early or begin later depending on the interactions between the ENSO-forced dynamics and internal variability [55]. While SAM decorrelates on 1-2-week time scales, an overall preference for either a positive or negative state can persist over extended periods; there is no seasonal locking of the SAM phase, although the circulation can amplify into the stratosphere during late-spring [56].

These spatial and temporal tendencies of climate modes combine to help explain the behaviour of the fire weather. From the analyses, the largest modulation of fire weather by the climate modes examined here occurs in eastern and south-eastern Australia, although much of Australia is affected to some degree. The key time of year for this influence is spring (SON), and to a lesser extent winter (JJA). Further work should focus on three-monthly moving averages to capture intra-seasonal relationships rather than focusing only on fixed seasons.

We suggest ENSO, and its extended time scale, set up the peak fire season, either directly or through the creation of favourable conditions for other climate modes to occur (i.e. correlations between different modes). Once established, the phase of ENSO sets a clear difference for the severity of the fire weather during the fire season. In the central and eastern parts of the country, differences between El Niño-like and La Niña-like conditions are large, persistent, and generally statistically significant. The phase of ENSO in JJA shows a widespread correlation with DJF FFDI90. The higher temperatures and reduced rainfall seen during El Niño-like conditions lets the drought factor build and increase the FFDI; as noted in [5], extended drought (at least 4–6 weeks) is a necessary requirement for the highest FFDI days to occur.

#### Regional differences in the dominant drivers of interannual variability

While ENSO is the primary precursor to extreme fire weather, the severity of any given fire season is further modulated by the IOD and/or the SAM. In some locations and seasons, these other sources of climate modulation are the primary driver, predominating over the ENSO effect. These effects vary depending on the region considered and the timing of the events. Where these other modes dominate was shown in Section 3.3 and Figs 10 and 11. In Summary:

**NSW Central Coast**. SAM is the dominant driver of fire weather in NSW during SON and DJF, with a negative SAM leading to an increase in FFDI, although the influence of ENSO remains strong in the region. The composite difference between positive and negative SAM is larger than that between the extremes of the ENSO3.4 SSTs. The central coast of NSW, as represented by Sydney and Williamtown in this dataset, is strongly affected. The years 1980, 2002 and 2013 are among the highest spring FFDI90 seasons in the region, and all characterised by strong negative SAM between July and November; only 2002 has a high ENSO3.4 SST. While there are few studies on fire and SAM for this region this strong relationship between FFDI and SAM corresponds with [9], finding of increase in rainfall associated with a positive SAM phase.

**VIC and southern SA**. All the climate modes examined here have some effect in this region, and a strong lag effect is observed. The FFDI90 during the peak fire season (DJF) is only weakly correlated with the concurrent climate variables; the strongest effect is seen with the SON variables. The results suggest higher ENSO3.4 SSTs in the spring set the stage for more extreme fire weather conditions during DJF in the region. This was also suggested in [21], who showed a relationship between spring ENSO indices and fire season fire activity. These conditions are exacerbated when a positive IOD is concurrently observed during spring, following [23]. This is indicated by the composite positive IOD FFDI90 values that are slightly higher compared to the highest composite ENSO values at Adelaide and Melbourne. Further, at Adelaide, the differences between positive and negative phases are only statistically significant with the IOD comparison. A negative lagged correlation with SAM is also identified in this region; the differences in DJF FFDI90 between positive and negative SAM in SON are only significant at Melbourne Airport station but show the same tendencies at Adelaide.

**TAS.** Similar to VIC and southern SA, all the climate modes examined here have some effect in this region. ENSO and IOD are dominant in winter and spring and the influence of ENSO continues into summer in the north of the state. These climate drivers also have a persistent effect from spring into summer. This corresponds with findings by [19] who identified a relationship between ENSO indices and fire activity in Tasmania. The influence of SAM is variable, with a positive relationship in winter and a negative effect in spring, this effect can be explained by rainfall variability as shown in [9]. Additionally, a negative lag relationship between spring SAM and summer FFDI is evident. However, these results differ to those found by [25] that suggests that the positive phase of SAM is related to increased fire activity One explanation for this discrepancy in findings is the different scope and methods used by [25], which focused on centennial scale charcoal records in western TAS and the phase of SAM over the preceding year. Our dataset lacks a high-quality station over western TAS, which has a different response to the climate drivers [9] and a different fire-weather climate [57] due to the orography in central TAS. Tasmania is complex, and further work on fire weather and fire activity there over varying temporal and spatial scales in relation to SAM and the other climate drivers should be pursued.

**Southern WA**. Further west, the correlations of FFDI90 and ENSO are weaker (and often not significant) in comparison to the eastern states, but still act in the same sense; lagged correlations are generally (slightly) stronger that the concurrent ones. The most significant correlations are with IOD, and this appears to be the dominant effect during JJA. The composite analysis from Perth reinforces these differences between positive and negative phases of IOD in SON and extending into DJF; the other climate modes examined apparently have minimal differences. This mixed result somewhat agrees with the findings of [9] that shows a mix of climate drivers dominating rainfall variability in southern WA in winter and spring.

**Northern Australia**. The correlations for Northern Australia are more difficult to interpret due to the fuel-limited fire regime there. In much of this region, the peak fire season is during JJA and SON and ends during DJF with the arrival of the monsoon/wet season. During these seasons, conditions are broadly favourable for fire, which is widespread. As noted earlier, the use of FFDI does not fully capture that relationship; it does capture the general severity of fire weather without taking fuel considerations into effect. Across much of QLD and NT, ENSO shows the strongest correlations at many locations. These are weaker and more spatially limited in JJA, but during SON the positive correlations are widespread across QLD and NT, and they extend into northern WA during DJF. A long lead time relationship (12+ months) between ENSO and fire *activity* has been identified. La Niña-like years bring enhanced rainfall, subsequently increasing fuel load and fire occurrence [18]. Negative correlations with SAM are observed mostly in QLD during SON. Cairns, Darwin and northern WA show some significant correlations with IOD, at both concurrent and lagged times. One interpretation of the DJF results for ENSO is that the positive correlations reflect a delayed onset on the Australian monsoon that often accompanies El Niño [58]. We suggest further work should consider analyses of the role of the Madden Julian Oscillation on fire weather variability, as this sub-seasonal climate driver plays a role in the timing of the monsoon and therefore rainfall variability in northern Australia [59].

#### Long term variability

In addition to the regional and interannual variability described earlier in the paper, there is clearly a long-term increase in fire weather over the 1973–2017 period examined in this study, as noted in [4] using earlier versions of the same dataset. As an all station average, the trend in standardized annual FFDI90 is positive and statistically significant at 0.24±0.13 points/decade (Figs 2 and 14). The trends in FFDI from the gridded FFDI dataset derived from [3] and shown in [26], although derived independently and covering a different time period, are in general agreement with those presented in this work. Spatially, the trends are positive across the continent and are observed during most seasons. Considered more carefully, the largest changes are occurring during spring and in the southern half of Australia. We assessed whether the apparent trend is primarily due any changes to the underlying climate drivers that influence fire weather; and found the trends in climate drivers appear to fall well short of explaining the overall trend. Therefore, the trends are examined in two different contexts:

1. The trend is primarily due to some natural decadal variability not explicitly considered here, mostly the IPO mentioned in the Introduction; and
2. The trend is a consequence of anthropogenic climate change resulting from the ~45% increase in greenhouse gas concentrations since the late-19th century [60].

In the first context, we consider decadal variability. A leading theory for understanding decadal variability of the climate is the Interdecadal Pacific Oscillation (IPO), the global manifestation of the northern hemisphere-only Pacific Decadal Oscillation [61]. Its associated SST pattern is similar to that of ENSO, but with a stronger signal in the extratropics [62]. Emerging evidence indicates that the IPO may influence the frequency of ENSO events, with three times more El Niño events during the positive phase, while La Niña is more prevalent during the negative phase [63]. Studies suggest that the rate of warming of the global mean surface temperature is faster during positive (El Niño-like) phases of the IPO [64,65]. Palmer et al. [66] suggest that it modulates drought in eastern Australia, with droughts more common during the positive phase, although they note this pattern has been broken over the most recent cycle. During the period examined here, only one cycle of the IPO has occurred, with the transitions identified from [67] marked on Fig 14. The IPO phase transitions do vaguely line up with the large changes in the all-station average annual FFDI90, with apparent decadal changes in the data circa 1980 and 2002. If the IPO were the main driving factor, then an 'oscillation' in FFDI90 would be expected, with an increase during the transition to the positive phase around 1978 and a decrease with the transition to the negative phase in 1998 as conditions became more La Niña-like and drought becomes less prevalent. The tendency of annual FFDI90 in Fig 14 does not correspond with the timing of the transitions, nor does the expected direction of the changes. Reliable records of fire weather that extend to periods before the 1970s are rare; one such record (for annual cumulative FFDI) from Canberra extending back to 1942 was presented in [17]. This time series suggests some variability on 10–20 years timescales (not shown here) but does not match this pattern or any of the other transition period. Together, these data suggest that the IPO is not a likely explanation for the changes that have been observed. There may well be decadal variability, but its source remains unclear.

The second context is that of anthropogenic climate change. The enhancement of greenhouse gases (GHGs) has been clearly linked to increased global temperatures and more frequent extreme temperatures, with Australia being no exception [60]. Climate projections for the 21st century unambiguously indicate higher fire weather for many parts of Australia [68]. By themselves, warmer average temperatures suggest increased fire weather. In addition to rising temperatures, changes to the large-scale mean meridional circulation and an expansion of the tropical belt driven in part by increasing GHGs [69] provide a possible mechanism for a long-term increases in fire weather, particularly in southern Australia. This poleward shift in the downward branch of the Hadley Cell changes large-scale rainfall patterns, generally drying the regions between 30 and 40° latitude. In the Southern Hemisphere, statistically significant tropical expansion from GHGs has been occurring from at least the late-1960s [70] with the greatest regional expansion over the Australia-East Asia corridor [71–73]. These expected changes in rainfall have similarities with patterns shown in observations [26]. Both warmer days and decreased southern rainfall should result in enhanced fire weather, in line with trends in fire weather (Fig 12); in northern Australia, trends are smaller or neutral, consistent with enhanced rainfall. The observed trends to date have generally been in excess of those projected from modelling studies [2,17,68,74].

This discussion here strongly points to anthropogenic climate change as one of the major causes of long-term variability in fire weather in Australia, particularly in the southern portions of the continent, consistent with conclusions based on gridded data throughout Australia [3] complementary to the approach of this study based on station data. However, this is not an attribution study, and the evidence presented here remains circumstantial. A formal attribution study of FFDI in southeastern Australia presented by [75] did indicate that an anthropogenic climate signal was detectable in the FFDI record, while [76] are less clear about the link. As suggested here, the magnitude of the anthropogenic signal in [75] was smaller than the swings suggested by ENSO. We also note that anthropogenic climate change may also impact fire weather indirectly, for example by modifying the underlying climate drivers. For example, a future increase in the frequency of El Niño events and positive IOD events [46,47] as well as increasing positive polarity of the SAM index [77] have all been suggested under global warming scenarios. Additionally, a recent study by [78] found that the trend and variability of Southern Hemisphere climate modes are (amongst others) being driven by climate change. The dynamics of global warming and its interactions with modes of interannual and decadal variability and its effect on fire weather in Australia remain uncertain.

## Summary and conclusion

This study explores the interannual and long-term variability of Australian fire weather. This is achieved through the analyses of the trends and variability of the seasonal 90^th^ percentile FFDI and ENSO, SAM and IOD and the relationship between these variables for 39 stations across Australia. Our key conclusions are:

- The Australian fire-weather climate shows a large degree of interannual variability and we hypothesise this is largely associated with the modulation of rainfall over the region.
- Spring (SON) is the season with the strongest correlations between 90^th^ percentile FFDI and the large-scale climate drivers for most regions.
- For much of the country, ENSO is the key driver for interannual variability. Seasons with higher NINO3.4 SSTs (i.e. El Niño-like) have more intense fire weather, while seasons with lower NINO3.4 SSTs (i.e. La Niña-like) can greatly reduce the fire weather danger. ENSO has the most impact in eastern and northern Australia. There are subtle differences in how the rest of the country is affected by these climate modes.
- The influence of IOD further modulates the fire weather on top of the ENSO signal. A positive IOD phase exacerbates effects of higher NINO 3.4 SST values. These often occur together (but not always). The largest effect is found in southeastern Australia.
- Negative SAM in SON can also enhance the effect of higher NINO3.4 SSTs but can also act independently (i.e. during lower NINO3.4 SSTs years), particularly in central coastal NSW.
- There is also a long-term upward trend in fire weather with the strongest trend found in southern Australia, in spring.
- This long-term trend is likely mostly due to anthropogenic climate change, rather than the influence of the IPO or the occurrence of congruent trends.
- Interannual changes are larger in magnitude that the longer-term changes that have occurred to date. Hence, the long-term signal can easily be overcome and result in a strong negative deviation from the general trend. An example of this is seen in the 2010–12 period, where a strong La Niña brought less intense fire weather to most of the country.

Understanding the interactions between climate drivers and Australian fire weather is a step towards improved seasonal forecasts of fire weather, potentially resulting in more effective fire planning and resource management. Furthermore, accepting that anthropogenic climate change is the most likely cause of the upward trend of fire weather in some parts of Australia will enable policy makers to make longer term decisions around managing fires in a changing climate.

## Supporting information

S1 Table Study periods for seasonal FFDI and weather data. (PDF)

S2 Table Years of the top and bottom 9 values of each index separated by season. (PDF)

S1 Fig Correlation coefficient values multiplied by 100 calculated for SON 90^th^ percentile FFDI and the preceding a. JJA NINO3.4 (one-season lag) b. MAM NINO3.4 (two-season lag). Significance greater than 99% in red, 95% in magenta and 90% green.
(PDF)

S2 Fig Correlation coefficient values multiplied by 100 calculated for JJA 90^th^ percentile FFDI and the preceding a. MAM NINO3.4 (one-season lag) b. DJF NINO3.4 (two-season lag). Significance greater than 99% in red, 95% in magenta and 90% green.
(PDF)

S3 Fig Correlation coefficient values multiplied by 100 calculated for MAM 90^th^ percentile FFDI and the preceding a. DJF NINO3.4 (one-season lag) b. SON NINO3.4 (two-season lag). Significance greater than 99% in red, 95% in magenta and 90% green.
(PDF)

S4 Fig Correlation coefficient values multiplied by 100 calculated for SON 90^th^ percentile FFDI and the preceding JJA DMI (one season lag) (1972–2017). Significance greater than 99% in red, 95% in magenta and 90% green.
(PDF)

S5 Fig Correlation coefficient values multiplied by 100 calculated for SON 90^th^ percentile FFDI and the preceding a. JJA SAM (one-season lag) b. MAM SAM (two-season lag) c. DJF 90^th^ percentile FFDI and the preceding JJA SAM (two-season lag). Significance greater than 99% in red, 95% in magenta and 90% green.
(PDF)

S6 Fig Correlation coefficient values multiplied by 100 calculated for JJA 90^th^ percentile FFDI and the preceding MAM SAM (one season lag) (1972–2017). Significance greater than 99% in red, 95% in magenta and 90% green.
(PDF)

S7 Fig Correlation coefficient values multiplied by 100 calculated for MAM 90^th^ percentile FFDI and the preceding DJF SAM (one season lag) (1972–2017). Significance greater than 99% in red, 95% in magenta and 90% green.
(PDF)

S8 Fig Partial correlation coefficient values multiplied by 100 for DJF 90^th^ percentile FFDI and a. DJF NINO3.4 b. DJF SAM (no season lag) (1972–2017). Significance greater than 99% in red, 95% in magenta and 90% green.
(PDF)

S9 Fig Partial correlation coefficient values multiplied by 100 for DJF 90^th^ percentile FFDI and a. SON NINO3.4 b. SON SAM c. SON IOD (one season lag) (1972–2017). Significance greater than 99% in red, 95% in magenta and 90% green.
(PDF)

S10 Fig Partial correlation coefficient values multiplied by 100 for SON 90^th^ percentile FFDI and a. SON NINO3.4 b. SON SAM c. SON IOD (no season lag) (1972–2017). Significance greater than 99% in red, 95% in magenta and 90% green.
(PDF)

S11 Fig Partial correlation coefficient values multiplied by 100 for JJA 90^th^ percentile FFDI and a. JJA NINO3.4 b. JJA SAM c. JJA IOD (no season lag) (1972–2017). Significance greater than 99% in red, 95% in magenta and 90% green.
(PDF)

S12 Fig Partial correlation coefficient values multiplied by 100 for MAM 90^th^ percentile FFDI and a. MAM NINO3.4 b. MAM SAM (no season lag) (1972–2017). Significance greater than 99% in red, 95% in magenta and 90% green.
(PDF)

S13 Fig Partial correlation coefficient values multiplied by 100 for DJF 90^th^ percentile FFDI and a. JJA NINO3.4 b. JJA SAM c. JJA IOD (two season lag) (1972–2017). Significance greater than 99% in red, 95% in magenta and 90% green.
(PDF)

S14 Fig Partial correlation coefficient values multiplied by 100 for JJA 90^th^ percentile FFDI and a. MAM NINO3.4 b. MAM SAM (one season lag) (1972–2017). Significance greater than 99% in red, 95% in magenta and 90% green.
(PDF)

S15 Fig Partial correlation coefficient values multiplied by 100 for MAM 90^th^ percentile FFDI and a. DJF NINO3.4 b. DJF SAM (one season lag) (1972–2017). Significance greater than 99% in red, 95% in magenta and 90% green.
(PDF)

S16 Fig Partial correlation coefficient values multiplied by 100 for MAM 90^th^ percentile FFDI and a. SON NINO3.4 b. SON SAM SON IOD (two season lag) (1972–2017). Significance greater than 99% in red, 95% in magenta and 90% green.
(PDF)

S17 Fig Partial correlation coefficient values multiplied by 100 for SON 90^th^ percentile FFDI and a. JJA NINO3.4 b. JJA SAM c. JJA IOD (one season lag) (1972–2017). Significance greater than 99% in red, 95% in magenta and 90% green.
(PDF)

S18 Fig Partial correlation coefficient values multiplied by 100 for SON 90^th^ percentile FFDI and a. MAM NINO3.4 b. MAM SAM (two season lag) (1972–2017). Significance greater than 99% in red, 95% in magenta and 90% green.
(PDF)
