# Supplementary material for: Understanding the variability of Australian fire weather between 1973 and 2017
Source: PLoS One. 2019 Sep 19;14(9):e0222328. doi: 10.1371/journal.pone.0222328 (PMC6752822; doi:10.1371/journal.pone.0222328)
Supplement: S1 Table — (PDF) [file pone.0222328.s001.pdf]

S1 Table. Study periods for seasonal FFDI and weather data

FFDI and weather	Number of samples	Date ranges
DJF FFDI90 and weather variables	45	December 1972-Feb 1973 to Dec 2016-February 2017
MAM FFDI90 and weather variables	45	March-May 1973 to March-May 2017
JJA FFDI90 and weather variables	44	June-August 1973 to June-August 2016
SON FFDI90 and weather variables	44	September-November 1973 to September-November 2016
