# Supplementary material for: Understanding the variability of Australian fire weather between 1973 and 2017
Source: PLoS One. 2019 Sep 19;14(9):e0222328. doi: 10.1371/journal.pone.0222328 (PMC6752822; doi:10.1371/journal.pone.0222328)
Supplement: S2 Table — (PDF) [file pone.0222328.s002.pdf]

S1 Table. Years of the top and bottom 9 values of each index separated by season

	ENSO (N34)		IOD (DMI)		SAM	
	top 9	Bottom 9	Top 9	Bottom 9	Top 9	Bottom 9
MAM	92, 83, 16, 98, 87, 15, 93,10, 05	11, 76, 00, 85, 08, 89, 75, 99, 74	--	--	82, 16, 98, 89, 93, 00, 99, 15, 74	07, 92, 77, 75, 86, 02, 80, 81, 90
JJA	15, 97, 87, 02, 91, 82, 04, 09, 94	78, 85,74, 98, 10, 99, 73, 75, 88	94, 83, 12, 97, 15, 76, 82, 11, 87	86, 73, 91, 85, 89, 16, 96, 80, 92	10, 79, 15, 98, 12, 93, 73, 04, 08	75, 81, 07, 96, 77, 92, 88, 74, 95
SON	15, 97, 82, 87, 02, 09, 91, 94, 86	83, 11, 99, 07, 98, 10, 88, 75, 73	97, 94, 06, 82, 15, 11, 02, 87, 12	73, 84, 81, 80, 98, 92, 74, 75, 96	10, 01, 99, 83, 85, 93, 08, 98, 92	73, 13, 96, 00, 94, 80, 97, 88, 02
DJF	16, 98, 83, 92, 73, 10, 87, 95, 03	96, 85, 11, 99, 08, 00, 76, 89, 74	--	--	00, 15, 08, 02, 99, 16, 74, 95, 12	01, 80, 87, 17, 83, 06, 75, 85, 77
