# Supplementary material for: Understanding the variability of Australian fire weather between 1973 and 2017
Source: PLoS One. 2019 Sep 19;14(9):e0222328. doi: 10.1371/journal.pone.0222328 (PMC6752822; doi:10.1371/journal.pone.0222328)
Supplement: S2 Fig — Correlation coefficient values multiplied by 100 calculated for JJA 90th percentile FFDI and the preceding a. MAM NINO3.4 (one-season lag) b. DJF NINO3.4 (two-season lag). Significance greater than 99% in red, 95% in magenta and 90% green. (PDF) [file pone.0222328.s004.pdf]

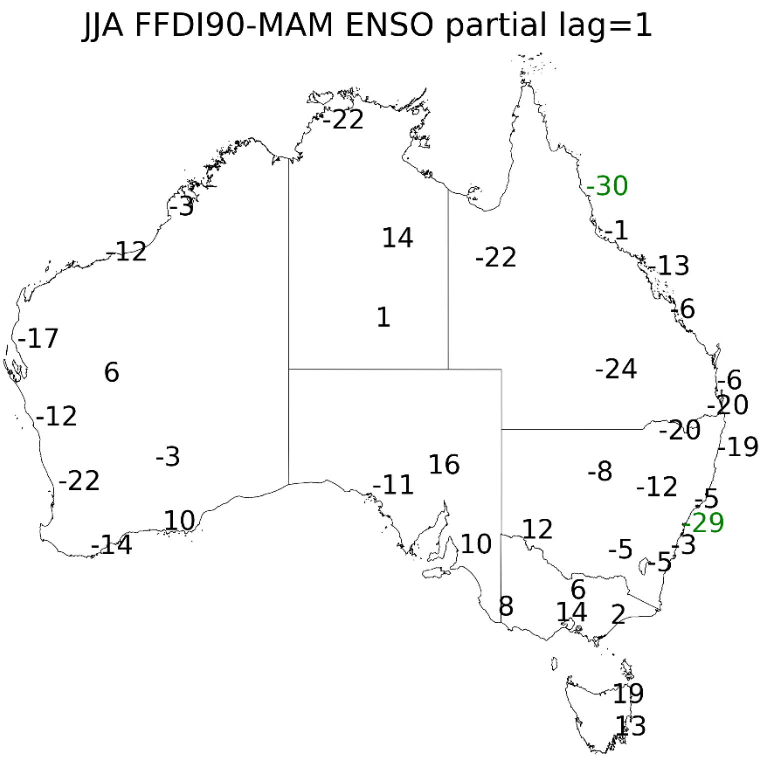

S2 Fig. Correlation coefficient values multiplied by 100 calculated for JJA 90th percentile FFDI and the preceding MAM NINO3.4 (one-season lag. Significance greater than 99% in red, 95% in magenta and 90% green.
