# Supplementary material for: Understanding the variability of Australian fire weather between 1973 and 2017
Source: PLoS One. 2019 Sep 19;14(9):e0222328. doi: 10.1371/journal.pone.0222328 (PMC6752822; doi:10.1371/journal.pone.0222328)
Supplement: S5 Fig — Correlation coefficient values multiplied by 100 calculated for SON 90th percentile FFDI and the preceding a. JJA SAM (one-season lag) b. MAM SAM (two-season lag) c. DJF 90th percentile FFDI and the preceding JJA SAM (two-season lag). Significance greater than 99% in red, 95% in magenta and 90% green. (PDF) [file pone.0222328.s007.pdf]

SON FFDI90-JJA SAM partial lag=1

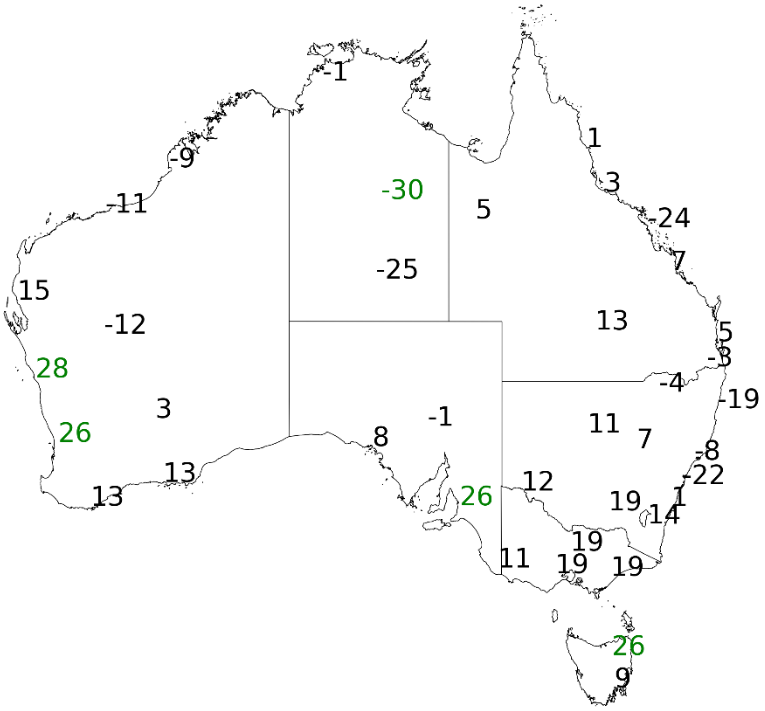

a.

SON FFDI90-MAM SAM partial lag=2

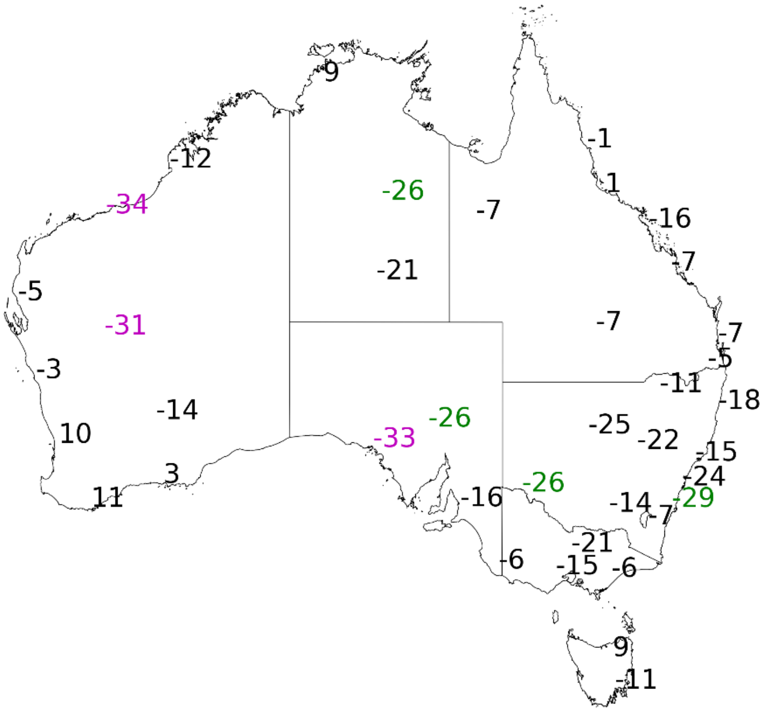

b.

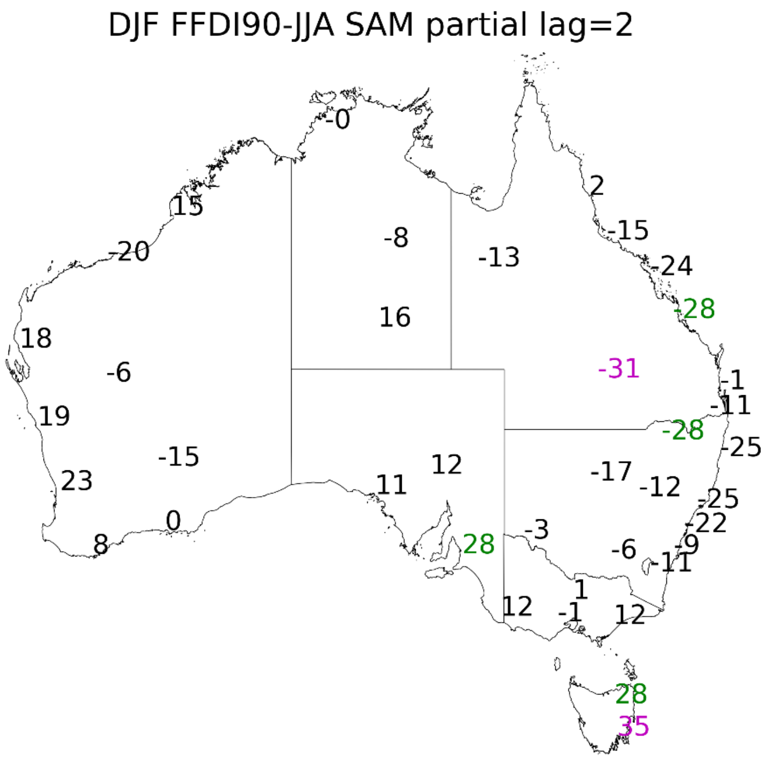

c.

S1 Fig. **Correlation coefficient values multiplied by 100 calculated for SON 90th percentile FFDI and the preceding a. JJA SAM (one-season lag) b. MAM SAM (two-season lag) c. DJF 90th percentile FFDI and the preceding JJA SAM (two-season lag). Significance greater than 99% in red, 95% in magenta and 90% green.**
