# Supplementary material for: Understanding the variability of Australian fire weather between 1973 and 2017
Source: PLoS One. 2019 Sep 19;14(9):e0222328. doi: 10.1371/journal.pone.0222328 (PMC6752822; doi:10.1371/journal.pone.0222328)
Supplement: S6 Fig — Significance greater than 99% in red, 95% in magenta and 90% green. (PDF) [file pone.0222328.s008.pdf]

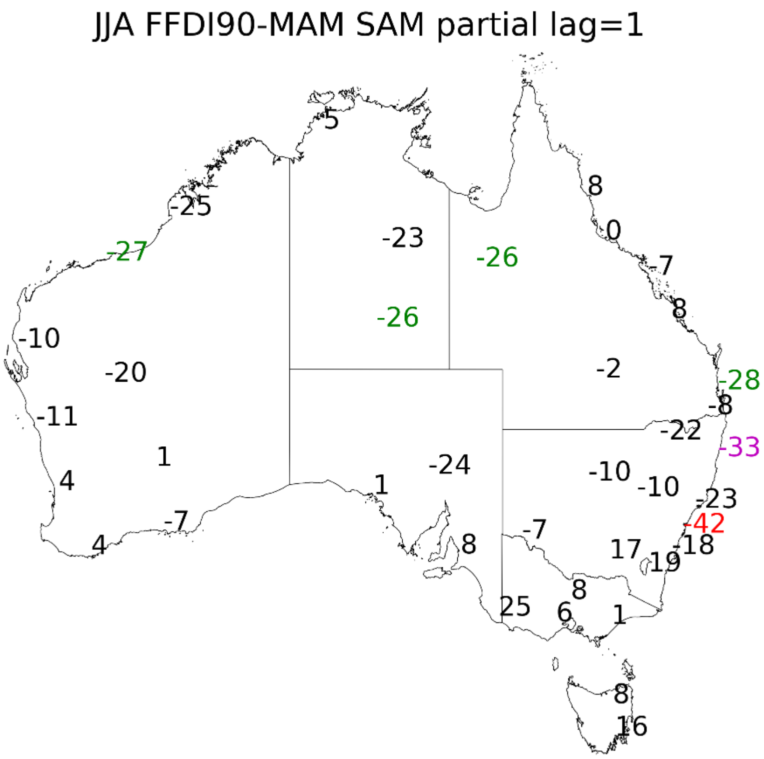


S6 Fig. Correlation coefficient values multiplied by 100 calculated for JJA 90th percentile FFDI and the preceding MAM SAM (one season lag) (1972 – 2017). Significance greater than 99% in red, 95% in magenta and 90% green.
