# Supplementary material for: Understanding the variability of Australian fire weather between 1973 and 2017
Source: PLoS One. 2019 Sep 19;14(9):e0222328. doi: 10.1371/journal.pone.0222328 (PMC6752822; doi:10.1371/journal.pone.0222328)
Supplement: S9 Fig — Partial correlation coefficient values multiplied by 100 for DJF 90th percentile FFDI and a. SON NINO3.4 b. SON SAM c. SON IOD (one season lag) (1972–2017). Significance greater than 99% in red, 95% in magenta and 90% green. (PDF) [file pone.0222328.s011.pdf]

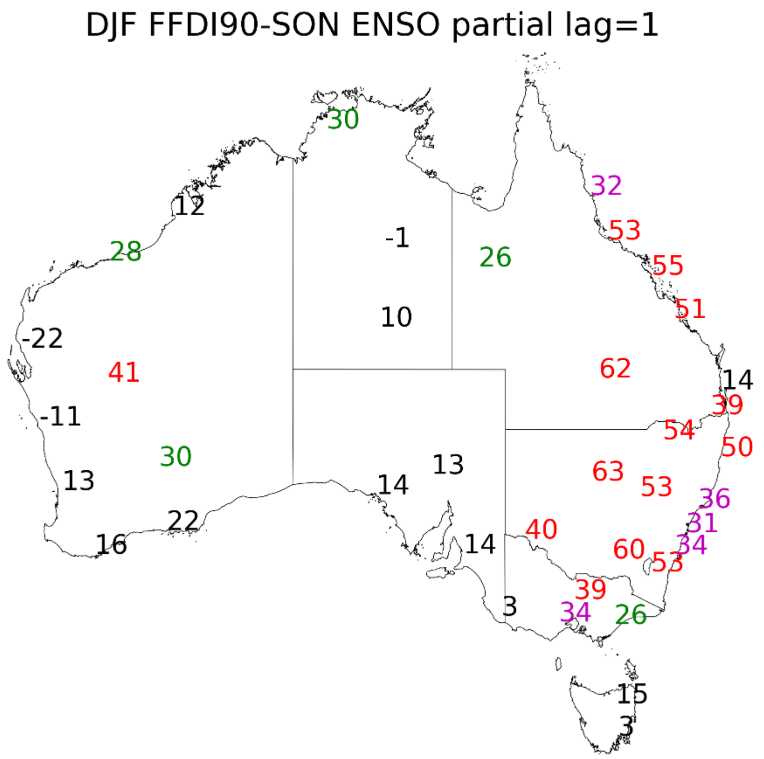
DJF FFDI90-SON ENSO partial lag=1
30
32
12
-1
53
28
26
55
51
10
-22
62
41
14
39
-11
54
50
30
13
63
13
14
53
36
22
31
14
40
16
34
60
53
39
3
34
26
15
3

a.

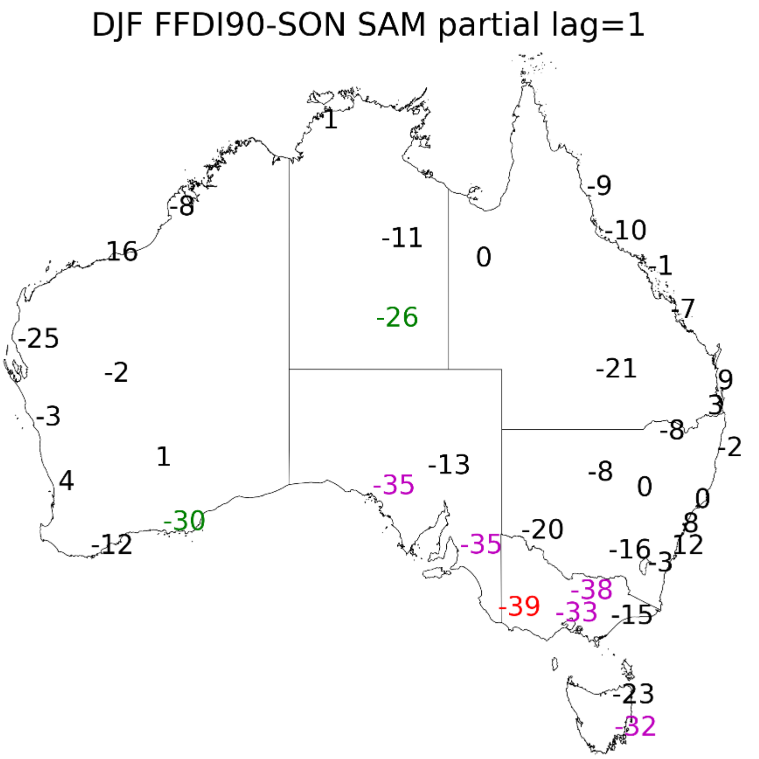

b.

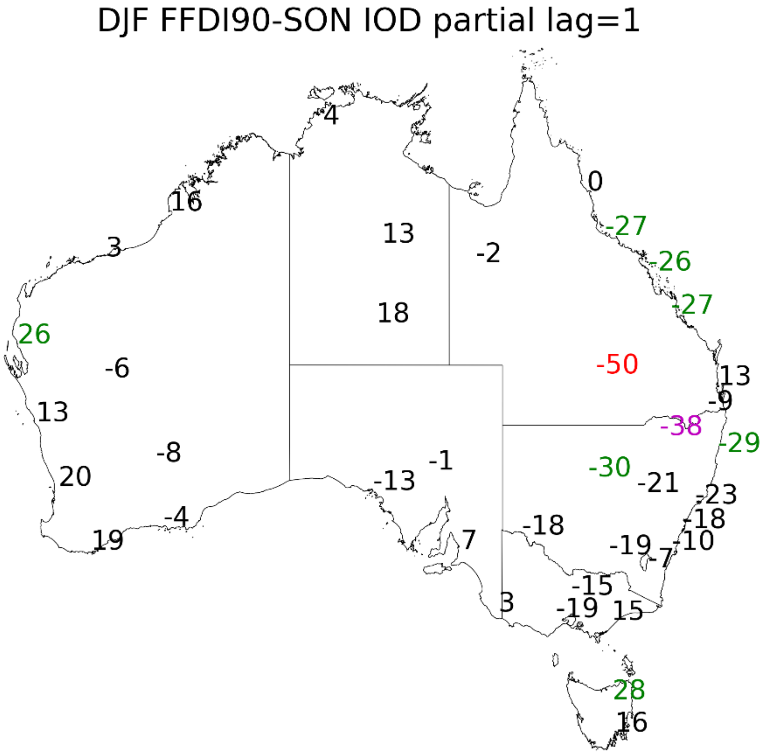

c.

S1 Fig. Partial correlation coefficient values multiplied by 100 for DJF 90th percentile FFDI and a. SON NINO3.4 b. SON SAM c. SON IOD (one season lag) (1972 – 2017). Significance greater than 99% in red, 95% in magenta and 90% green.
