# Supplementary material for: Understanding the variability of Australian fire weather between 1973 and 2017
Source: PLoS One. 2019 Sep 19;14(9):e0222328. doi: 10.1371/journal.pone.0222328 (PMC6752822; doi:10.1371/journal.pone.0222328)
Supplement: S10 Fig — Partial correlation coefficient values multiplied by 100 for SON 90th percentile FFDI and a. SON NINO3.4 b. SON SAM c. SON IOD (no season lag) (1972–2017). Significance greater than 99% in red, 95% in magenta and 90% green. (PDF) [file pone.0222328.s012.pdf]

SON FFDI90-SON NINO3.4 partial lag=0

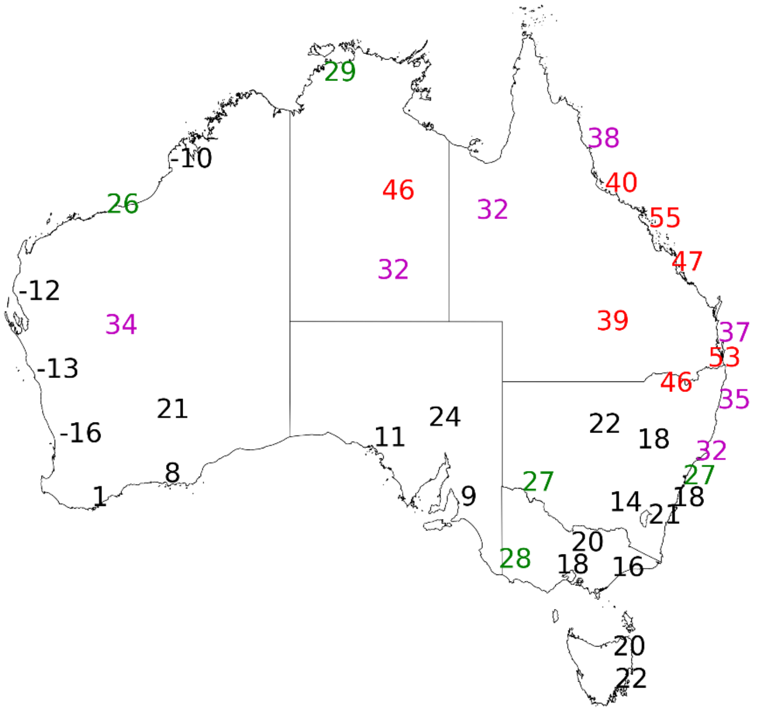

a.

SON FFDI90-SON SAM partial lag=0

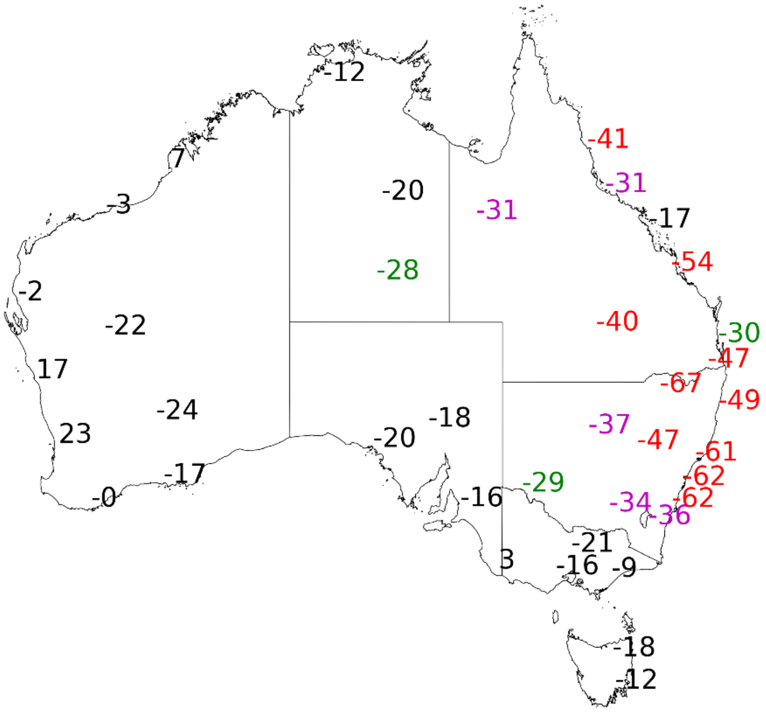

b.

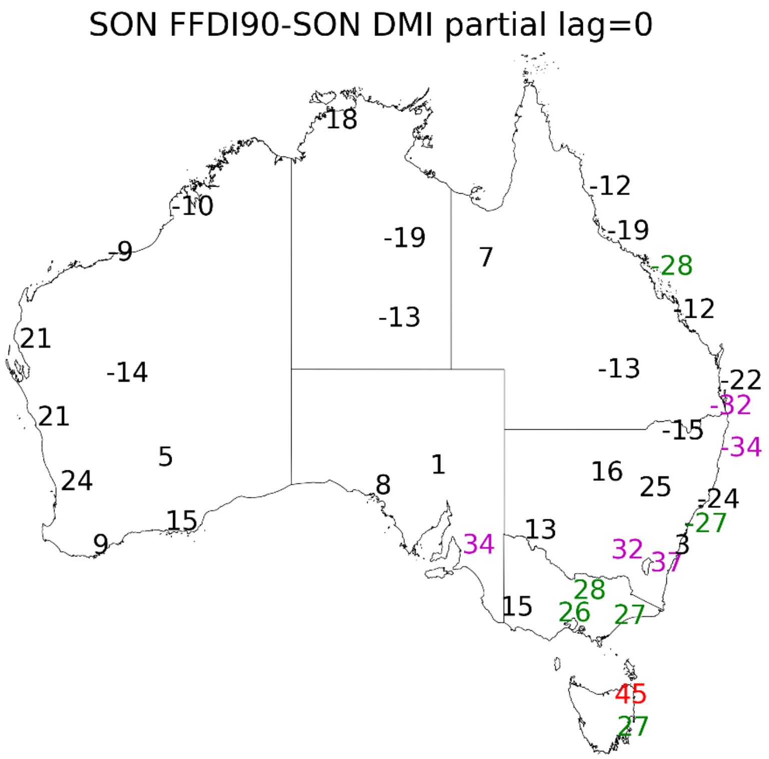

c.

S1 Fig. Partial correlation coefficient values multiplied by 100 for SON 90th percentile FFDI and a. SON NINO3.4 b. SON SAM c. SON IOD (no season lag) (1972 – 2017). Significance greater than 99% in red, 95% in magenta and 90% green.
