# Supplementary material for: Understanding the variability of Australian fire weather between 1973 and 2017
Source: PLoS One. 2019 Sep 19;14(9):e0222328. doi: 10.1371/journal.pone.0222328 (PMC6752822; doi:10.1371/journal.pone.0222328)
Supplement: S11 Fig — Partial correlation coefficient values multiplied by 100 for JJA 90th percentile FFDI and a. JJA NINO3.4 b. JJA SAM c. JJA IOD (no season lag) (1972–2017). Significance greater than 99% in red, 95% in magenta and 90% green. (PDF) [file pone.0222328.s013.pdf]

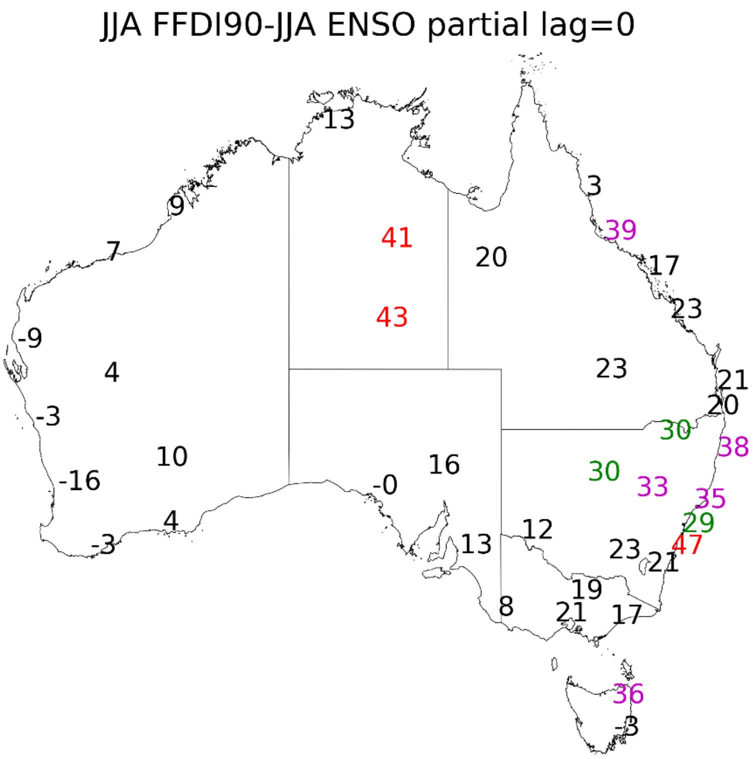
JJA FFDI90-JJA ENSO partial lag=0
13
9
7
-9
4
-3
10
-16
4
-3
41
43
-0
16
20
3
39
17
23
23
21
20
30
38
30
33
35
29
47
12
13
23
21
19
8
21
17
36
-3

a.

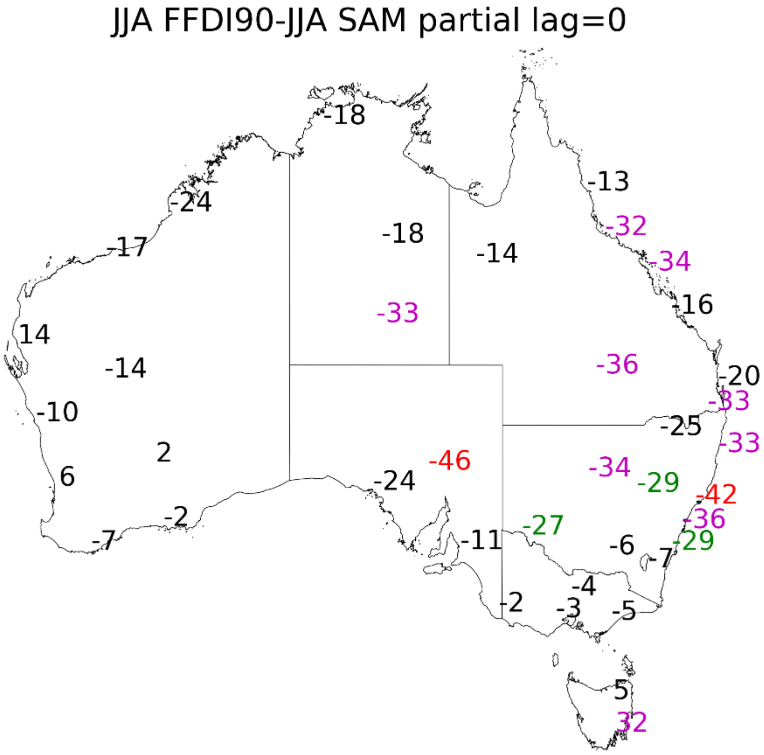
JJA FFDI90-JJA SAM partial lag=0
-18
-24
-17
14
-14
-10
2
6
-2
-7
-18
-33
-14
-13
-32
-34
-16
-36
-20
-33
-25
-33
-46
-24
-34
-29
-42
-36
-29
-27
-11
-6
-7
-4
-2
-3
-5
5
32

b.

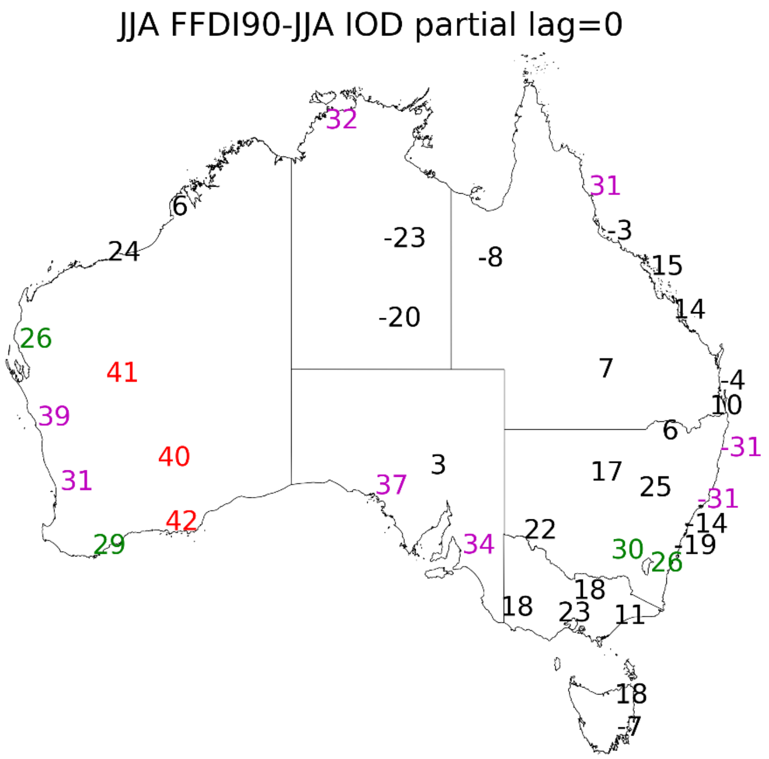

c.

S1 Fig. Partial correlation coefficient values multiplied by 100 for JJA 90th percentile FFDI and a. JJA NINO3.4 b. JJA SAM c. JJA IOD (no season lag) (1972 – 2017). Significance greater than 99% in red, 95% in magenta and 90% green.
