# Supplementary material for: Understanding the variability of Australian fire weather between 1973 and 2017
Source: PLoS One. 2019 Sep 19;14(9):e0222328. doi: 10.1371/journal.pone.0222328 (PMC6752822; doi:10.1371/journal.pone.0222328)
Supplement: S13 Fig — Partial correlation coefficient values multiplied by 100 for DJF 90th percentile FFDI and a. JJA NINO3.4 b. JJA SAM c. JJA IOD (two season lag) (1972–2017). Significance greater than 99% in red, 95% in magenta and 90% green. (PDF) [file pone.0222328.s015.pdf]

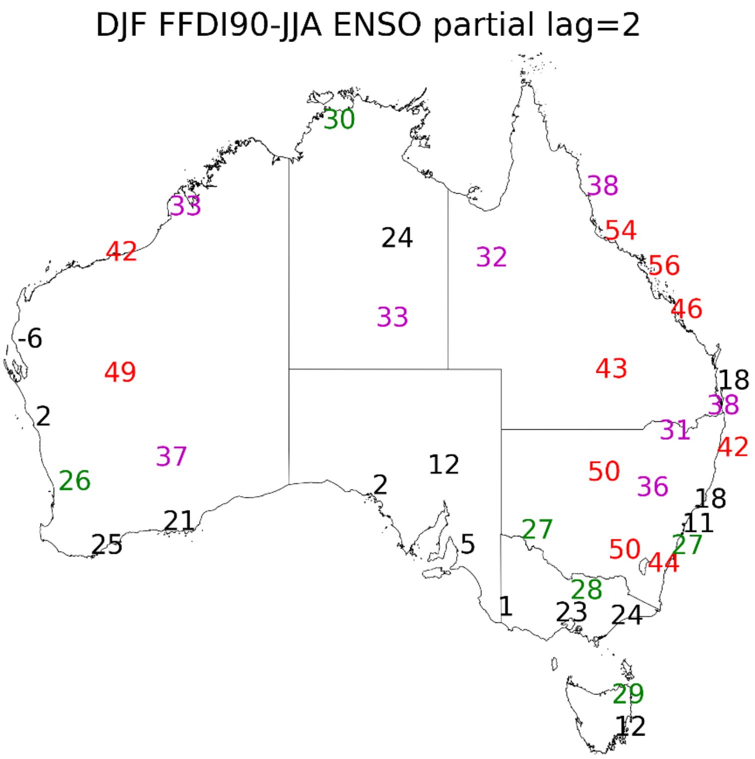
DJF FFDI90-JJA ENSO partial lag=2
30
38
33
24
32
54
56
42
46
33
-6
49
43
18
38
2
31
42
37
12
26
2
50
36
18
21
5
27
-11
25
50
27
44
28
1
23
24
29
12

a.

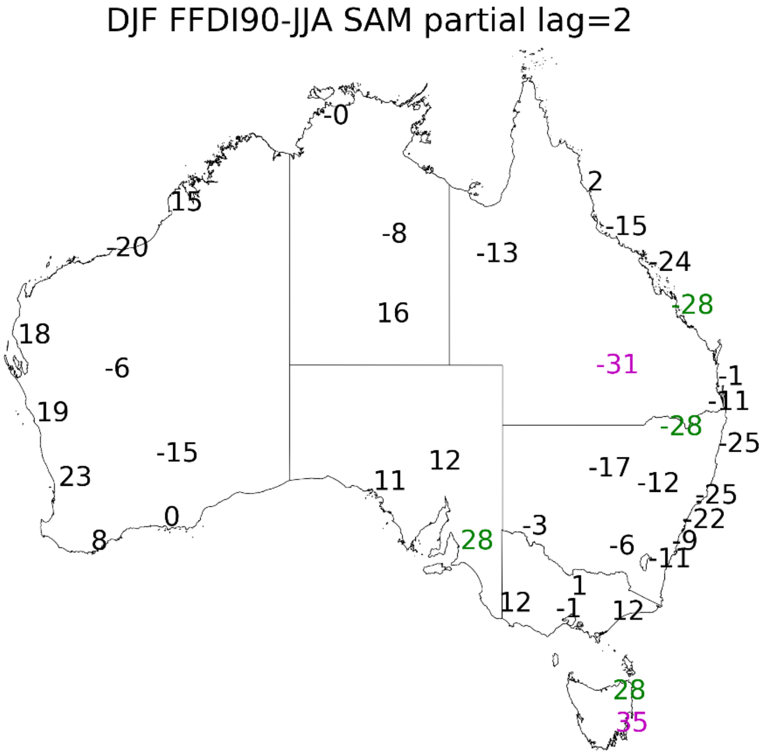
DJF FFDI90-JJA SAM partial lag=2
-0
2
15
-15
-8
-20
-13
-24
-28
16
18
-6
-31
-1
-11
19
-28
-25
-15
12
-17
23
11
-12
-25
0
-22
28
-3
8
-6
-9
-11
1
12
-1
12
28
35

b.

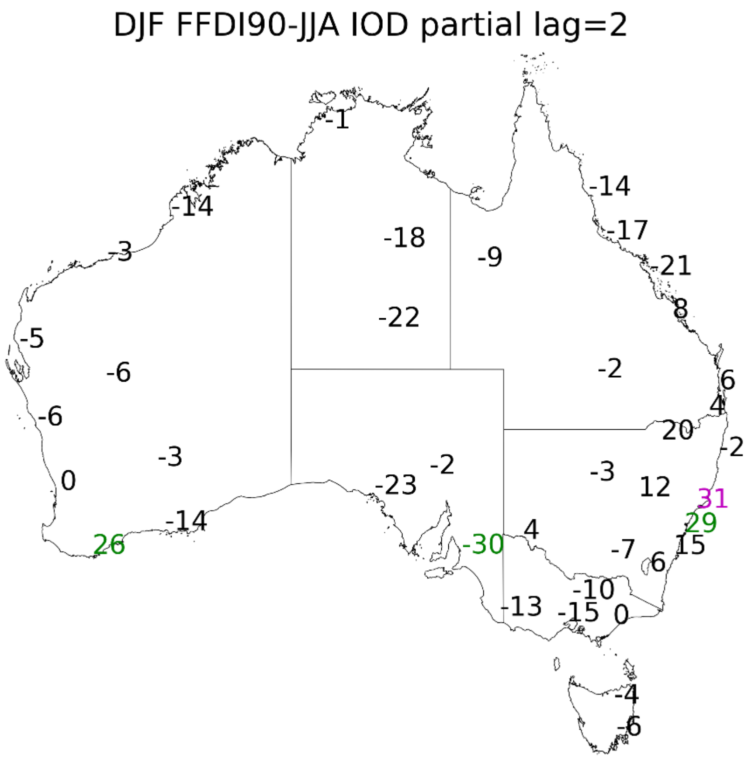

c.

S1 Fig. Partial correlation coefficient values multiplied by 100 for DJF 90th percentile FFDI and a. JJA NINO3.4 b. JJA SAM c. JJA IOD (two season lag) (1972 – 2017). Significance greater than 99% in red, 95% in magenta and 90% green.
