# Supplementary material for: Understanding the variability of Australian fire weather between 1973 and 2017
Source: PLoS One. 2019 Sep 19;14(9):e0222328. doi: 10.1371/journal.pone.0222328 (PMC6752822; doi:10.1371/journal.pone.0222328)
Supplement: S16 Fig — Partial correlation coefficient values multiplied by 100 for MAM 90th percentile FFDI and a. SON NINO3.4 b. SON SAM SON IOD (two season lag) (1972–2017). Significance greater than 99% in red, 95% in magenta and 90% green. (PDF) [file pone.0222328.s018.pdf]

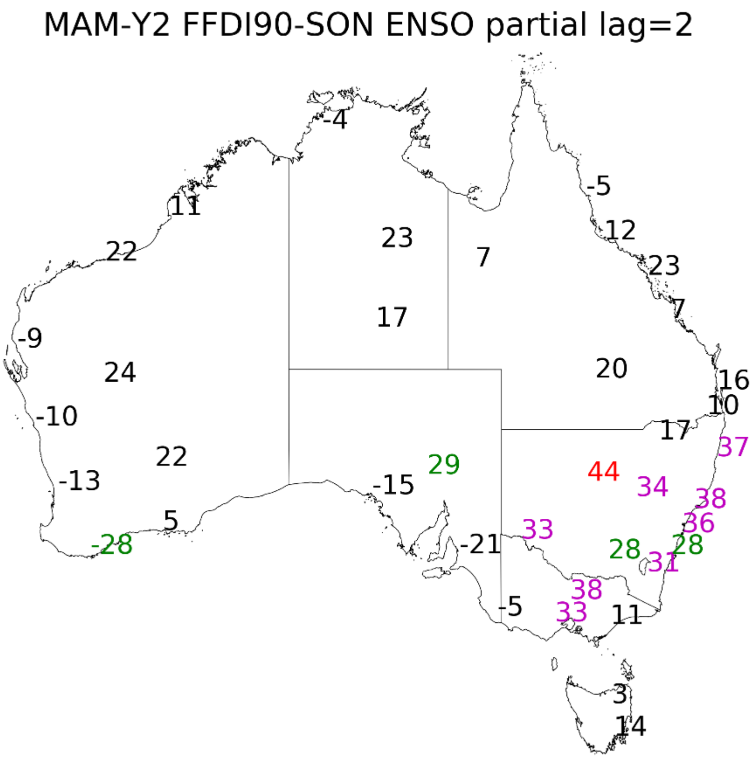
MAM-Y2 FFDI90-SON ENSO partial lag=2
-4
-5
11
12
23
22
23
7
7
17
-9
20
24
16
10
-10
17
37
22
29
44
-13
-15
34
38
5
36
33
-21
-28
28
28
31
38
-5
33
11
3
14

a.

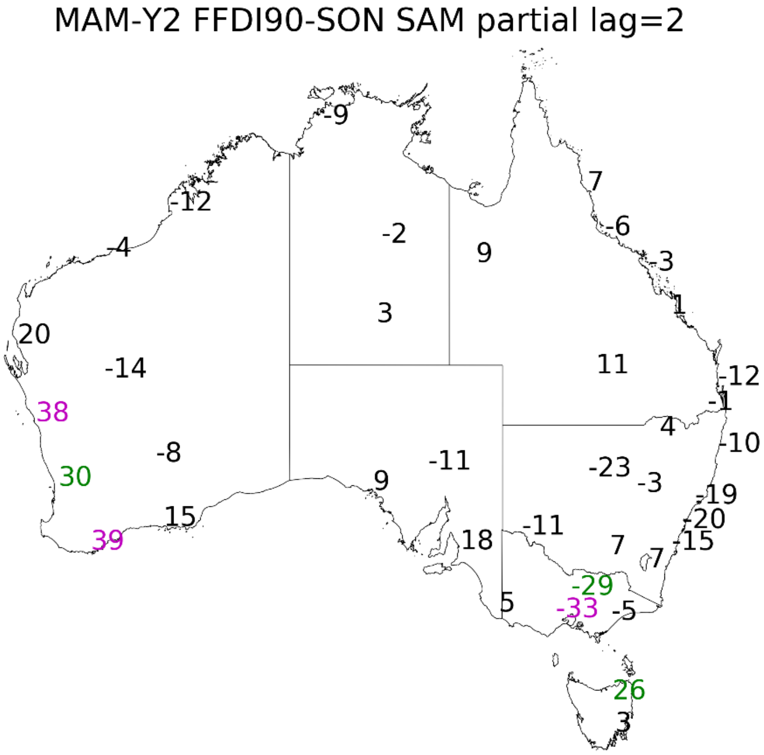
MAM-Y2 FFDI90-SON SAM partial lag=2
-9
7
-12
-6
-4
-2
-3
9
1
3
20
11
-14
-12
-1
38
4
-10
-8
-11
-23
30
9
-3
-19
-20
15
-11
39
18
-15
7
7
-29
5
-33
-5
26
3

b.

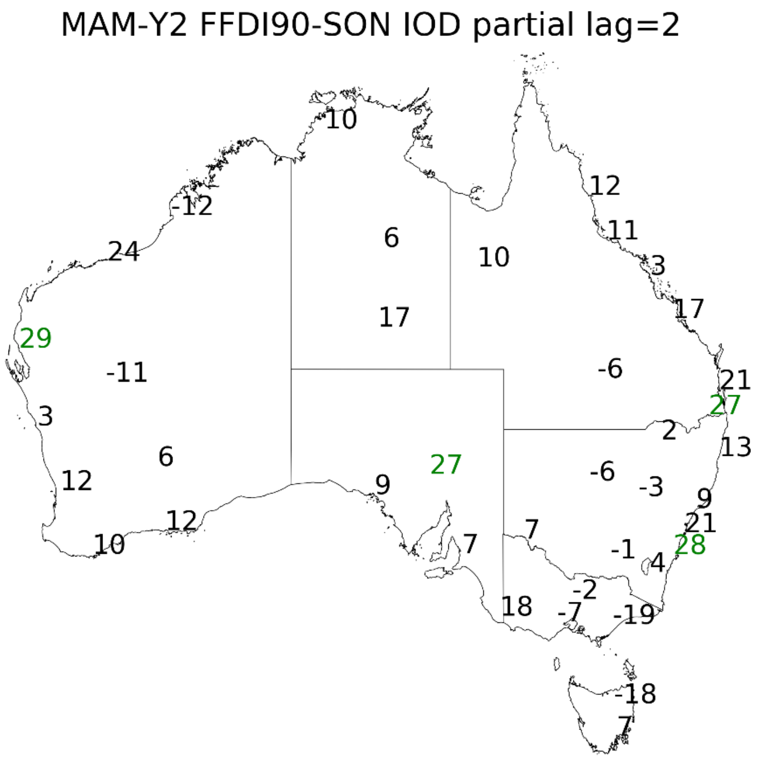

c.

S1 Fig. Partial correlation coefficient values multiplied by 100 for MAM 90th percentile FFDI and a. SON NINO3.4 b. SON SAM SON IOD (two season lag) (1972 – 2017). Significance greater than 99% in red, 95% in magenta and 90% green.
