# Supplementary material for: Understanding the variability of Australian fire weather between 1973 and 2017
Source: PLoS One. 2019 Sep 19;14(9):e0222328. doi: 10.1371/journal.pone.0222328 (PMC6752822; doi:10.1371/journal.pone.0222328)
Supplement: S17 Fig — Partial correlation coefficient values multiplied by 100 for SON 90th percentile FFDI and a. JJA NINO3.4 b. JJA SAM c. JJA IOD (one season lag) (1972–2017). Significance greater than 99% in red, 95% in magenta and 90% green. (PDF) [file pone.0222328.s019.pdf]

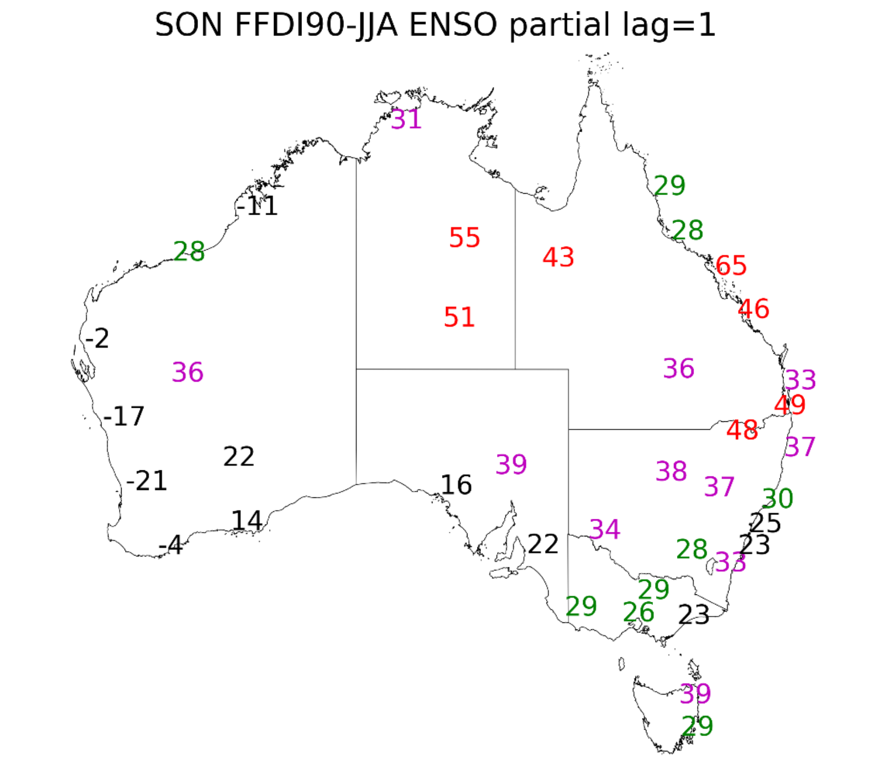
SON FFDI90-JJA ENSO partial lag=1
31
29
-11
28
55
43
28
65
46
51
-2
36
36
33
49
-17
48
37
22
39
38
37
-21
16
30
14
25
34
-4
22
28
23
33
29
29
26
23
39
29

a.

b.

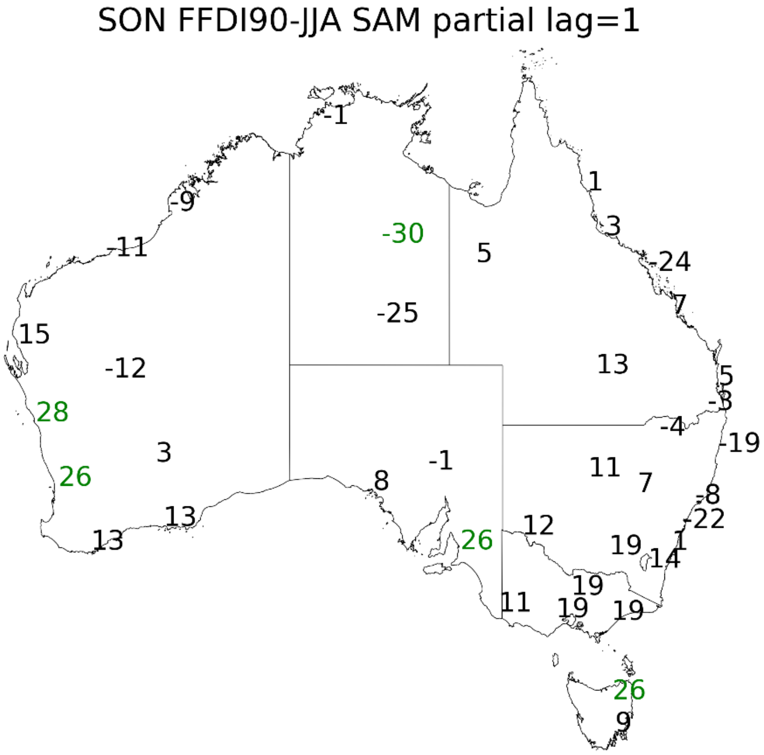
SON FFDI90-JJA SAM partial lag=1
-1
1
-9
3
-11
-30
5
-24
7
-25
15
13
-12
5
-3
28
-4
-19
3
-1
11
8
26
7
-8
-22
13
12
26
19
1
13
14
19
11
19
19
26
9

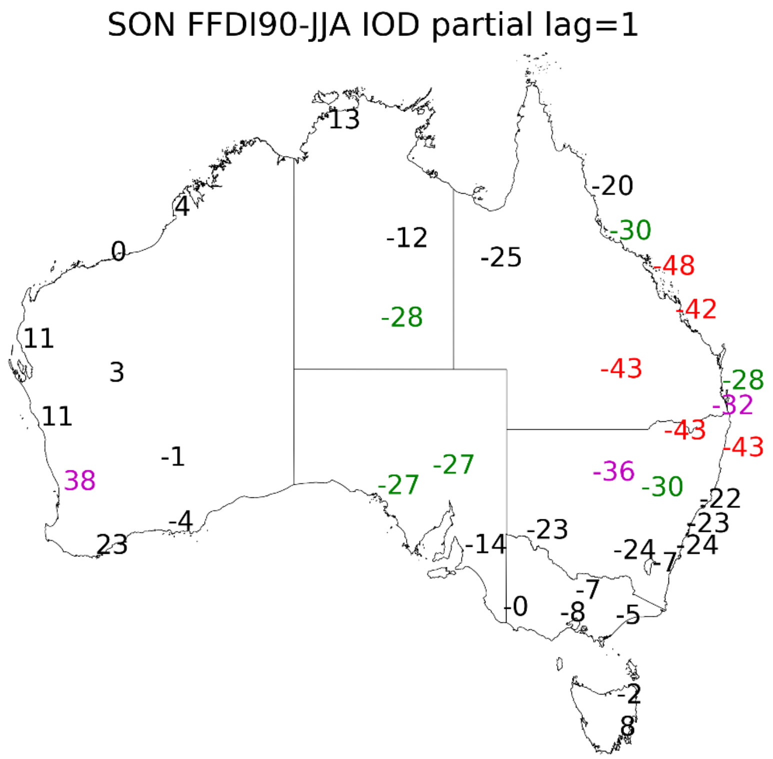

c.

S1 Fig. Partial correlation coefficient values multiplied by 100 for SON 90th percentile FFDI and a. JJA NINO3.4 b. JJA SAM c. JJA IOD (one season lag) (1972 – 2017). Significance greater than 99% in red, 95% in magenta and 90% green.
