# Supplementary material for: Understanding the variability of Australian fire weather between 1973 and 2017
Source: PLoS One. 2019 Sep 19;14(9):e0222328. doi: 10.1371/journal.pone.0222328 (PMC6752822; doi:10.1371/journal.pone.0222328)
Supplement: S18 Fig — Partial correlation coefficient values multiplied by 100 for SON 90th percentile FFDI and a. MAM NINO3.4 b. MAM SAM (two season lag) (1972–2017). Significance greater than 99% in red, 95% in magenta and 90% green. (PDF) [file pone.0222328.s020.pdf]

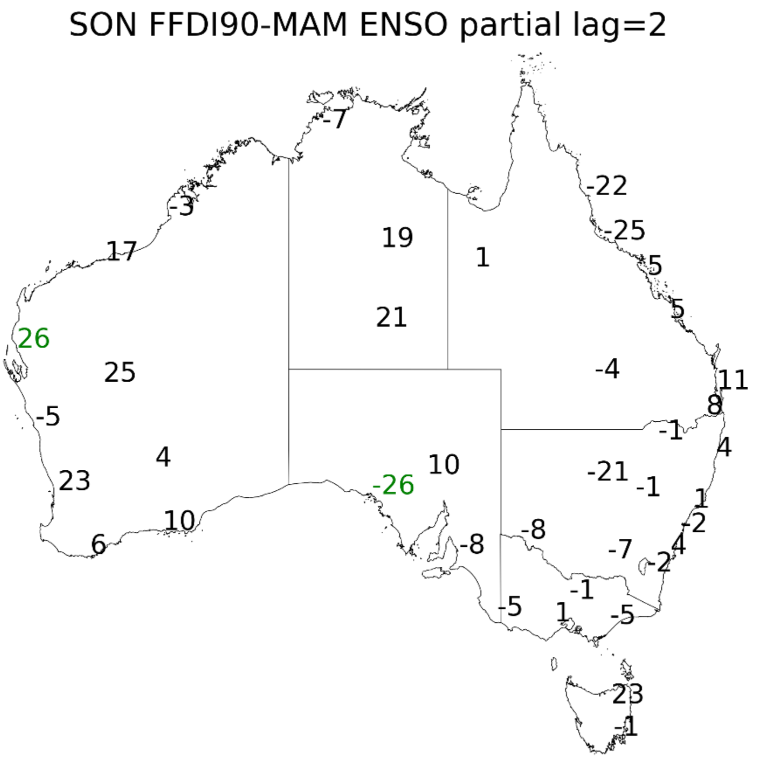

a.

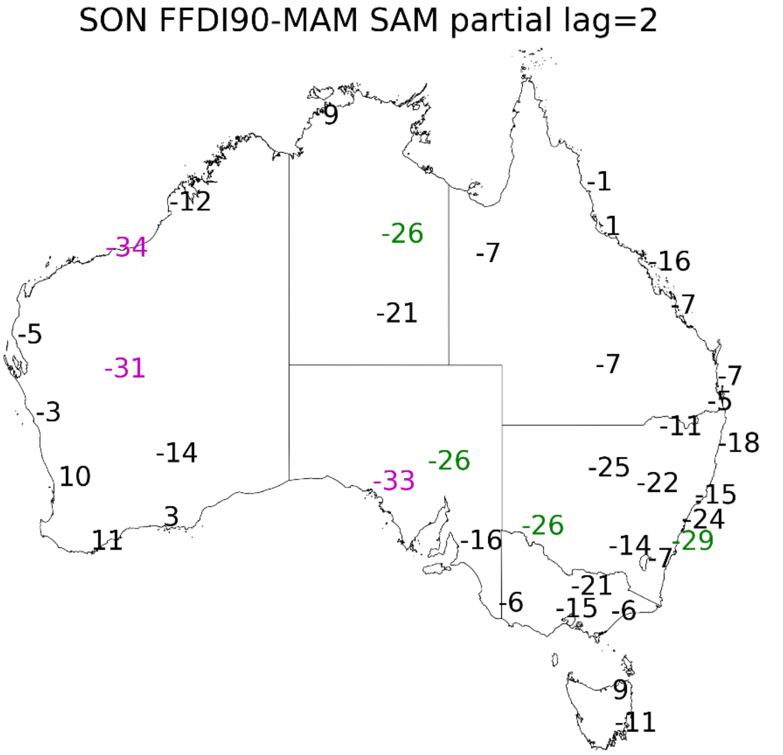

b.

S1 Fig. Partial correlation coefficient values multiplied by 100 for SON 90th percentile FFDI and a. MAM NINO3.4 b. MAM SAM (two season lag) (1972 – 2017). Significance greater than 99% in red, 95% in magenta and 90% green.
